# Akkermansia muciniphila Ameliorates Acetaminophen-Induced Liver Injury by Regulating Gut Microbial Composition and Metabolism

**DOI:** 10.1128/spectrum.01596-21

**Published:** 2022-02-02

**Authors:** Jiafeng Xia, Longxian Lv, Boqiang Liu, Shuting Wang, Sitong Zhang, Zhengjie Wu, Liya Yang, Xiaoyuan Bian, Qiangqiang Wang, Kaicen Wang, Aoxiang Zhuge, Shengjie Li, Ren Yan, Huiyong Jiang, Kaijin Xu, Lanjuan Li

**Affiliations:** a State Key Laboratory for Diagnosis, National Clinical Research Center for Infectious Diseases, The First Affiliated Hospital, School of Medicine, Zhejiang University, Hangzhou, China; b Collaborative Innovation Center for Diagnosis and Treatment of Infectious Diseases, Hangzhou, China; c Zhejiang Provincial Key Laboratory of Laparoscopic Technology, Sir Run Run Shaw Hospital, School of Medicine, Zhejiang University, Hangzhou, China; d Department of General Surgery, The Second Affiliated Hospital, School of Medicine, Zhejiang University, Hangzhou, China; National Institutes of Health

**Keywords:** probiotic, *Akkermansia*, liver injury, microbiota, transcriptome, short-chain fatty acids

## Abstract

The gut microbiota drives individual sensitivity to excess acetaminophen (APAP)-mediated hepatotoxicity. It has been reported that the bacterium Akkermansia muciniphila protects hosts against liver disease via the liver-gut axis, but its therapeutic potential for drug-induced liver injury remains unclear. In this study, we aimed to investigate the effect of A. muciniphila on APAP-induced liver injury and the underlying mechanism. Administration of A. muciniphila efficiently alleviated APAP-induced hepatotoxicity and reduced the levels of serum alanine aminotransferase (ALT) and aspartate transaminase (AST). A. muciniphila significantly attenuated APAP-induced oxidative stress and the inflammatory response, as evidenced by restoration of the reduced glutathione/oxidized glutathione (GSH/GSSG) balance, enhanced superoxide dismutase (SOD) activity, reduced proinflammatory cytokine production, and alleviation of macrophage and neutrophil infiltration. Moreover, A. muciniphila maintained gut barrier function, reshaped the perturbed microbial community and promoted short-chain fatty acid (SCFA) secretion. The beneficial effects of A. muciniphila were accompanied by alterations in hepatic gene expression at the transcriptional level and activation of the phosphatidylinositol 3-kinase (PI3K)/Akt signaling pathway. Our results suggested that A. muciniphila could be a potential pretreatment for APAP-induced liver injury.

**IMPORTANCE** Our work revealed that A. muciniphila attenuated APAP-induced liver injury by alleviating oxidative stress and inflammation in the liver, and its hepatoprotective effect was accompanied by activation of the PI3K/Akt pathway and mediated by regulation of the composition and metabolic function of the intestinal microbiota. This finding suggested that the microbial community is a non-negligible impact on drug metabolism and probiotic administration could be a potential therapy for drug-induced liver injury.

## INTRODUCTION

Acetaminophen (APAP) is a frequently used nonsteroidal antipyretic analgesic drug in clinical treatment. At therapeutic doses, APAP is mainly metabolized by phase II conjugating enzymes such as UDP-glucuronosyltransferases (UGTs) and sulfotransferases (SULTs) and converted to nontoxic soluble compounds (1). However, overdose of APAP has potent hepatotoxicity and is the major cause of acute liver injury worldwide. After the nontoxic metabolic pathway reaches saturation, the cytotoxic compound N-acetyl-p-benzoquinone imine (NAPQI) is generated from the excess APAP via catalysis by cytochrome P450 (CYP) family enzymes and depletes glutathione (GSH) in the liver (2). Hepatocytes cannot detoxify NAPQI when GSH is exhausted, and the remaining NAPQI attempts to combine with mitochondrial proteins, leading to oxidative stress and mitochondrial dysfunction and eventually to hepatocyte necrosis (3, 4).

Trillions of mutualistic bacteria belonging to more than 1,000 species colonize the human gastrointestinal tract and are defined as the gut microbiota (5). The gut microbiota maintains a symbiotic relationship with its host. Changes in the pathological condition in the host are often accompanied by dynamic variability in the microbial profile, while the microbiota, in turn, participates in disease development. An increasing number of studies have reported the important association between the gut microbiota and liver disease. Through contributing to bile acid circulation, endotoxin translocation, modulation of Kupffer cells via Toll-like receptors, gut-derived T cell migration and other effects, microbiota dysbiosis is strongly related to liver diseases such as nonalcoholic fatty liver disease (NAFLD), autoimmune liver disease, and cirrhosis (6–8). Modulation of microbial structure has become an important treatment method to improve the outcomes of liver diseases. Although it has been reported that the microbiota mediates APAP hepatotoxicity and that selected species protect against drug-induced liver damage, the impact of the microbiota on APAP-induced liver injury and its mechanism are insufficiently studied (9). Only a few probiotics have been confirmed to be hepatoprotective against APAP-induced liver injury in animal studies.

Akkermansia muciniphila is a strictly anaerobic Gram-negative species belonging to *Verrucomicrobia*, which constitutes of 0.5%∼5% of the human intestinal microflora (10). A. muciniphila is considered a highly promising probiotic due to its representative benefits in promoting immunomodulatory and metabolic functions and maintaining the host’s homeostatic condition. A. muciniphila adheres to the mucus layer and consumes mucus glycan oligosaccharides as a nutrient (11). The degradation metabolites of mucin, such as short-chain fatty acids (SCFAs), can regulate the expression of genes related to lipid metabolism and the immune response. A large number of studies have demonstrated that A. muciniphila is an important factor in pathological progression. Reduced abundance of A. muciniphila leads to a thinner mucous layer and impaired intestinal barrier integrity (12). A. muciniphila abundance is negatively correlated with bowel symptoms in inflammatory bowel disease (IBD) patients (13). A. muciniphila administration significantly ameliorated immune-mediated liver injury in a concanavalin A (ConA) model (14).

This study was designed to explore the effect of A. muciniphila on APAP-induced liver injury and investigate the underlying mechanisms through multiomic integration, incorporating 16S sequencing, transcriptomics, and metabolomics.

## RESULTS

### A. muciniphila ameliorated APAP-induced liver injury.

**FIG 1:**
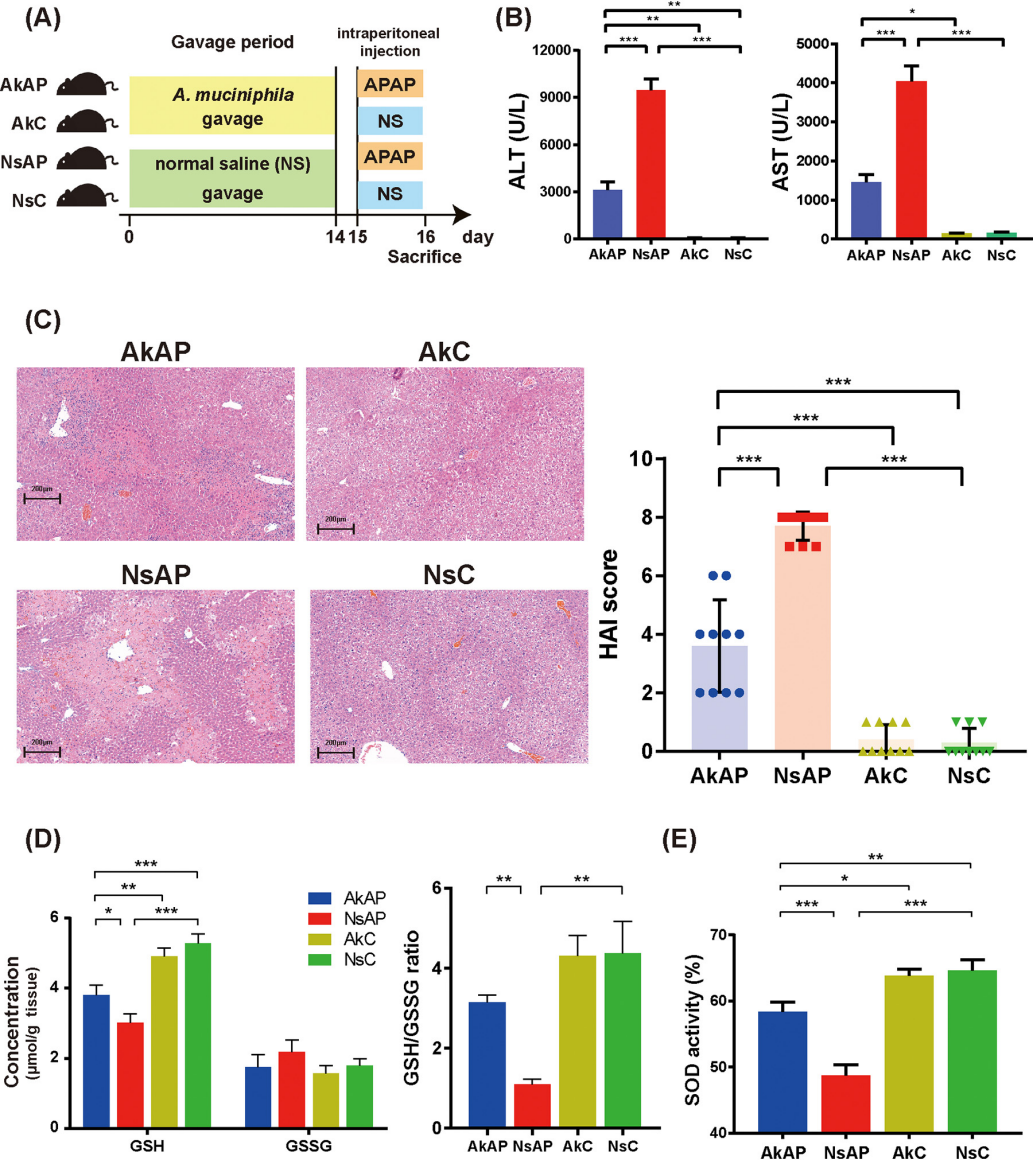
Administration of A. muciniphila ameliorated APAP-induced liver injury and oxidative stress. (A) Schematic diagram of the design of the animal experiment. (B) Serum levels of ALT and AST. (C) Representative liver H&E staining (left panel, original magnification × 20, scale bar = 200 μm) and liver pathological HAI (right panel). The data are presented as the mean ± SEM. (D) Reduced GSH level, GSSG level, and GSH/GSSG ratio. (E) SOD activity. *, *P < *0.05; **, *P < *0.01; and ***, *P < *0.001 for the comparison. AkAP, A. muciniphila+APAP; AkC, A. muciniphila+saline control; NsAP, normal saline+APAP; NsC, normal saline+saline control; *n* = 10 each group.

A mouse model of acute liver injury model was established at 24 h after 300 mg/kg APAP dosing. As typical biological markers of liver function, serum alanine aminotransferase (ALT), and aspartate transaminase (AST) levels were determined to evaluate the severity of liver injury. The ALT and AST levels were significantly elevated after intraperitoneal injection of APAP. Moreover, administration of A. muciniphila (AkAP) markedly decreased ALT and AST levels compared with those in the group treated with normal saline + APAP (NsAP) (Fig. 1B). Hematoxylin and eosin (H&E) staining analysis revealed signs of extensive necrosis and massive inflammatory cell infiltration in the NsAP group. Only sporadic small necrotic foci and relatively low inflammatory cell infiltration observed in the group treated with A. muciniphila + APAP (AkAP) (Fig. 1C). No obvious pathological findings were detected in the group treated with A. muciniphila + saline control (AkC) and that treated with normal saline + saline control (NsC). Consistent with the changes in ALT and AST levels, the modified histological activity index (HAI) presented a similar tendency: the HAI score was significantly increased in NsAP compared with NsC and was partially restored after A. muciniphila administration (Fig. 1C). Taken together, the above results indicated that A. muciniphila effectively attenuated APAP-induced liver injury.

### A. muciniphila alleviated APAP-induced oxidative stress and the inflammatory response.

The increased mitochondrial permeability and oxidative stress caused by intracellular GSH depletion are considered the initial but critical triggers of APAP-induced degeneration (15). We evaluated GSH, oxidized glutathione (GSSG), and superoxide dismutase (SOD) levels to assess vulnerability to oxidative stress. As shown in Fig. 1D and E, 24 h after APAP treatment, the GSH, GSH/GSSG, and SOD levels were substantially reduced. However, AkAP exhibited evident restoration of those indicators compared with NsAP. In addition, the GSSG levels were not significantly different among the four groups.

**FIG 2:**
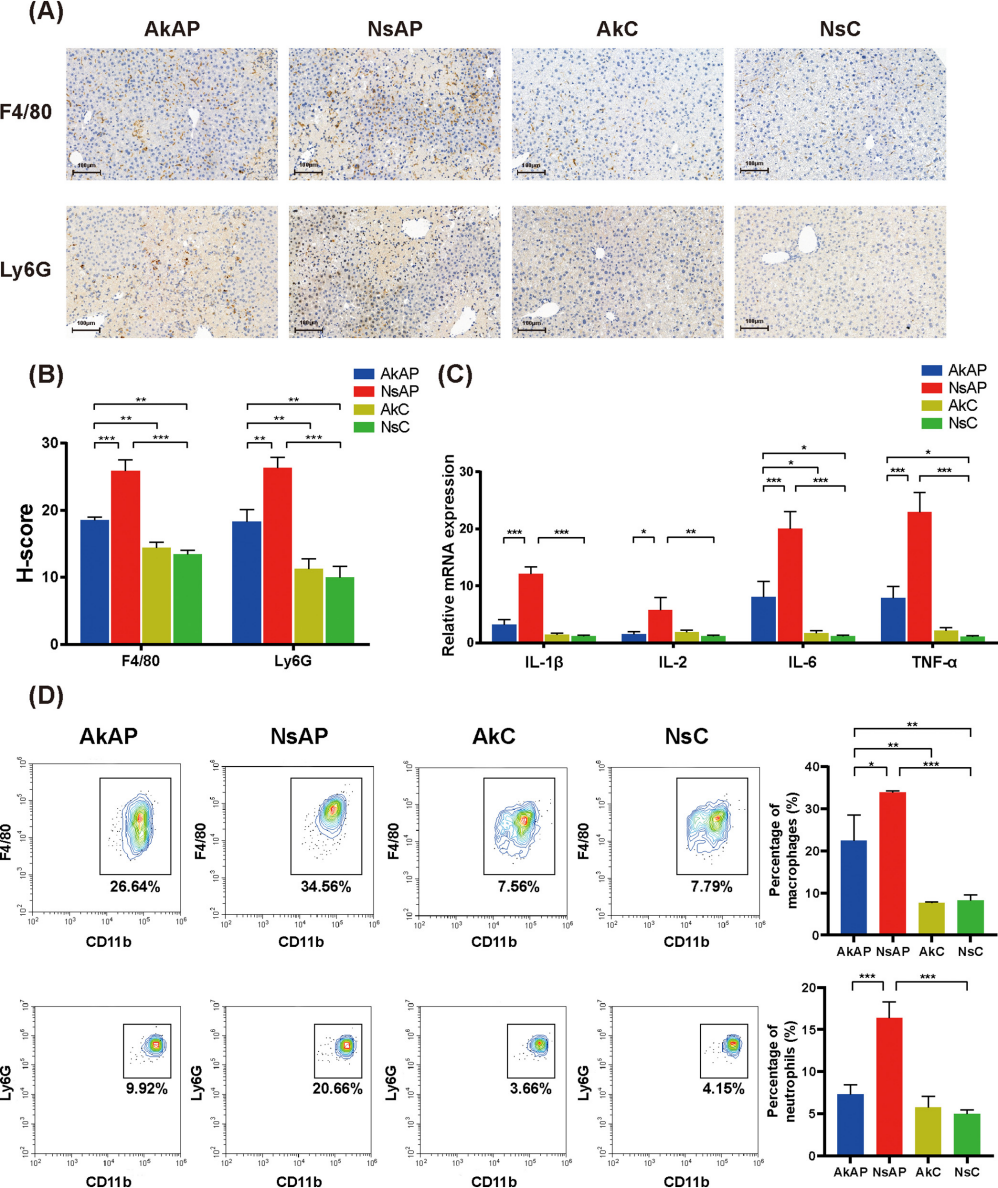
A. muciniphila improved APAP-induced oxidative stress and the inflammatory response in the liver. (A) Representative images of immunohistochemical staining of F4/80 and Ly6G (original magnification × 40, scale bar = 100 μm). (B) H-score of immunohistochemical staining. The H-score was assessed by ImageJ, demonstrating the staining intensity. (C) Relative hepatic mRNA expression of proinflammatory cytokines. (D) Representative flow cytometric plots of macrophages and neutrophils and quantitative analysis of cell percentage (*n* = 4 each group). The data are presented as the mean ± SEM. *, *P < *0.05; **, *P < *0.01; and ***, *P < *0.001 for the comparison. AkAP, A. muciniphila+APAP; AkC, A. muciniphila+saline control; NsAP, normal saline+APAP; NsC, normal saline+saline control; *n* = 6 each group for immunohistochemical analysis; *n* = 10 each group for RT-qPCR; *n* = 4 each group for flow cytometry.

The inflammatory response mediated by activated Kupffer cells and infiltrated immune cells plays a crucial role in APAP toxicity cascade development (1). To evaluate hepatic inflammation, we detected the alterations in the expression of representative inflammatory markers (IL-1β, IL-2, IL-6, IL-10, TNF-α, and IFN-γ) at the transcriptional level. Mice in NsAP showed evidently lower expression of IL-1β, IL-2, IL-6, and TNF-α than those in NsC, demonstrating that APAP resulted in an inflammatory response in the liver. However, A. muciniphila attenuated inflammation, as indicated by the downregulation of these cytokines in AkAP compared with NsAP (Fig. 2C). IL-10 and IFN-γ showed no noticeable difference among the four groups (Fig. S1). Moreover, immunohistochemical analysis with anti-F4/80 or anti-Ly6G was performed to estimate the hepatic infiltration of macrophages and neutrophils. As expected, the staining results indicated that the recruitment of macrophages and neutrophils was markedly promoted by APAP, while A. muciniphila evidently reduced macrophage and neutrophil infiltration (Fig. 2A and B). Flow cytometric analysis also confirmed increased accumulation of macrophages and neutrophils in AkAP compared with NsC, while AkAP showed lower numbers of macrophages and neutrophils compared with NsAP (Fig. 2D). Besides, we observed that other immune cell subsets (CD4^+^ T cell, CD8^+^ T cell, B cell, NK cell, NKT cell) had no significant variation (Fig. S2). These results revealed that A. muciniphila improved APAP hepatotoxicity-induced oxidative stress and the inflammatory response in the liver.

### A. muciniphila modulated the alteration of hepatic transcriptional expression during APAP challenge.

**FIG 3:**
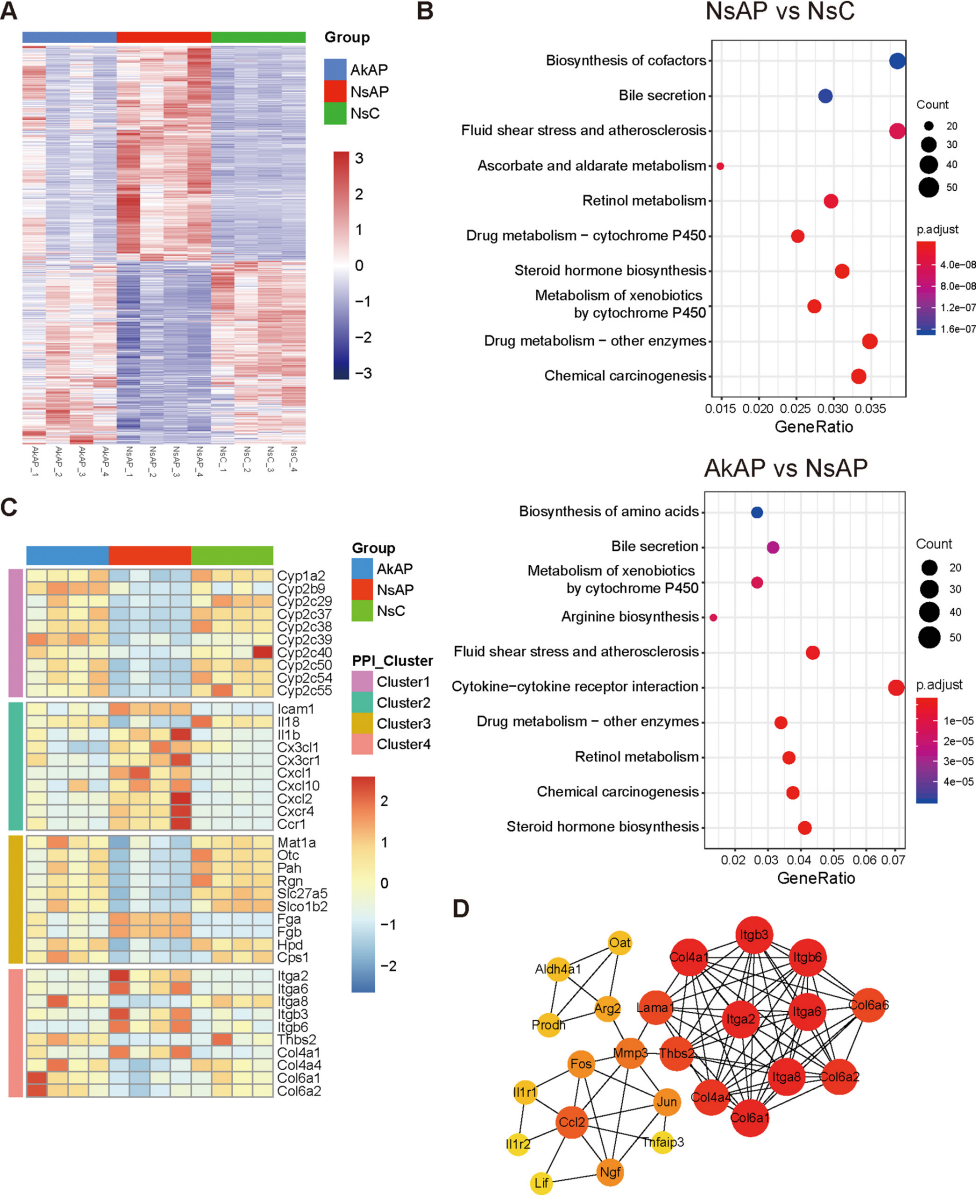
A. muciniphila intervention affected hepatic transcriptomic alteration. (A) Heatmap of DEGs in AkAP versus NsAP or NsAP versus NsC. (B) KEGG enrichment analyses of DEGs between the comparisons (upper, NsAP versus NsC; lower, AkAP versus NsAP). (C) Heatmap of the top 10 hub genes from four clusters of the PPI network. (D) Visualization of the cluster 4 network. AkAP, A. muciniphila+APAP; NsAP, normal saline+APAP; NsC, normal saline+ saline control; *n* = 3 each group.

To further investigate how A. muciniphila contributed to liver protection from APAP-induced injury, we performed transcriptomic analysis of livers harvested from AkAP, NsAP, and NsC (*n* = 4 per group). In the comparison between NsAP and NsC, 2810 genes were identified as differentially expressed genes (DEGs) by using the criteria *P* < 0.05 and |log_2_ (fold change)| > 1. Among these DEGs, 1,558 and 1,252 were significantly upregulated and downregulated, respectively, in NsAP compared with NsC. In the comparison between AkAP and NsAP, 838 and 846 DEGs were significantly upregulated and downregulated, respectively, in NsAP. Fig. 3A shows an intuitive visualization of the hierarchical clustering of DEGs in the two comparisons. NsAP and NsC had apparently distinct gene expression patterns, whereas the clustering patterns of AkAP and NsC were relatively similar and homogeneous, indicating the improvement of the pathological process. We identified 777 DEGs that were promoted by APAP injection but suppressed by A. muciniphila administration and 711 DEGs that were suppressed by APAP injection but promoted by A. muciniphila administration through overlap analysis of all DEGs from the two transcriptomic comparisons, suggesting that A. muciniphila markedly modulated the transcriptional alteration caused by APAP. Kyoto Encyclopedia of Genes and Genomes (KEGG) enrichment analysis of the DEGs from the comparison of NsAP versus NsC revealed 73 pathways based on the criterion adj-*P* <0.05. The affected pathways were mainly assigned to drug metabolism, involving CYPs, UGTs, SULTs, and the glutathione *S*-transferase (GST) family (Fig. 3B). KEGG enrichment analysis of the DEGs from the comparison of AkAP versus NsAP identified 50 pathways, such as steroid hormone biosynthesis, metabolism of xenobiotics by cytochrome P450 and cytokine-cytokine receptor interaction. A protein-protein interaction (PPI) network was constructed by mapping genes from these 50 significant pathways to classify genes participating in hepatoprotection. Four predominant clusters were extracted from the PPI network, representing drug metabolism, inflammation, amino acid biosynthesis, and signaling conduction (the top 10 hub genes of each cluster are shown in Fig. 3C, cluster 1–3 are shown in Fig. S3, cluster 4 is shown in Fig. 3D, and all hub genes are shown in the Table S3). Notably, the phosphatidylinositol 3-kinase (PI3K)/Akt pathway was identified as the most enriched pathway in cluster 4. These results suggested that the protection by A. muciniphila could be attributed to modulation of transcriptional expression related to metabolism, inflammation, and signal transduction.

### A. muciniphila activated the PI3K/Akt pathway and inhibited apoptosis in the liver.

**FIG 4:**
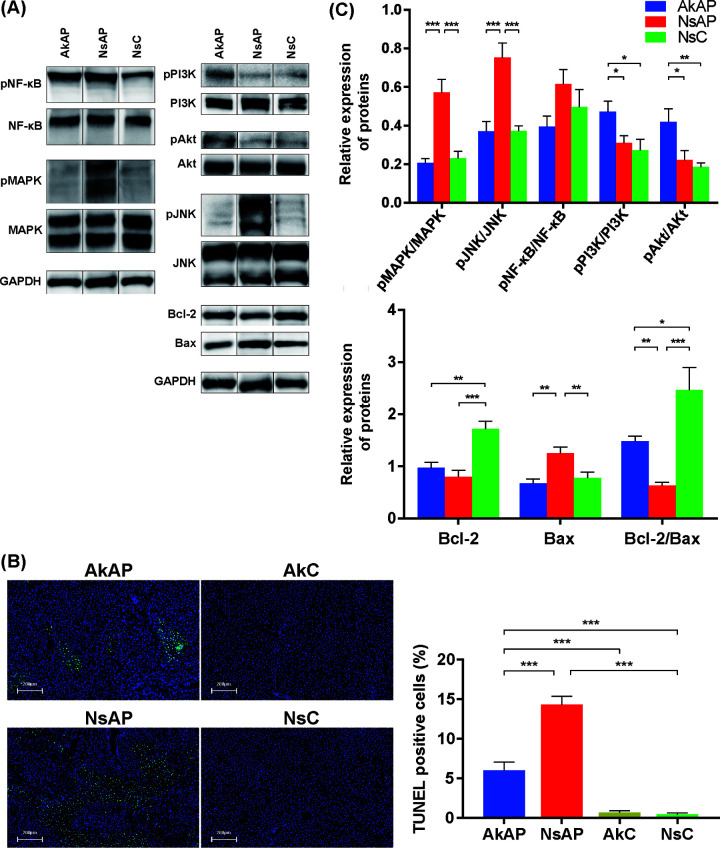
A. muciniphila intervention inhibited APAP-induced apoptosis and activated the PI3K/Akt signaling pathway. (A) Representative Western blot bands. (B) Representative liver DAPI/TUNEL immunofluorescence staining (upper panel, original magnification × 20, scale bar = 200 μm) and percentage of TUNEL-positive cells in each group (lower panel). (C) Quantitative analysis of protein expression. The data are presented as the mean ± SEM. *, *P < *0.05; **, *P < *0.01; and ***, *P < *0.001 for the comparison. AkAP, A. muciniphila+APAP; AkC, A. muciniphila+saline control; NsAP, normal saline+APAP; NsC, normal saline+saline control; *n* = 6 each group.

The transcriptomic results showed that the PI3K/Akt pathway might be responsible for the effects of A. muciniphila under APAP challenge. The activated PI3K/Akt pathway inhibits cellular apoptosis via modulation of Bcl-2 and Bax (16). APAP treatment had no influence on Akt and PI3K phosphorylation, but A. muciniphila promoted the phosphorylation of Akt and PI3K after APAP challenge (Fig. 4A and C). The protein expression of Bax in NsAP was strikingly higher than that in AkAP and NsC, and Bcl-2 expression in AkAP was significantly higher than that in NsAP, with no noticeable alteration compared with NsC. As an important indicator of apoptosis, the Bcl-2/Bax ratio was decreased in NsAP, and A. muciniphila tended to reverse this change to the level observed in NsC. Activation of c-Jun N-terminal kinase (JNK) and downstream proteins acts as a critical mediator amplifying mitochondrial dysfunction and triggering apoptosis. We also found that A. muciniphila prevented the APAP-elicited enhancement in the phosphorylation levels of ERK1/2 and JNK without a prominent effect on the phosphorylation level of NF-κB. Consistent with these findings, the terminal deoxynucleotidyltransferase-mediated dUTP nick end labeling (TUNEL) assay demonstrated a reduced percentage of TUNEL-positive hepatocytes in AkAP compared with NsAP, indicating that A. muciniphila reduced nuclear DNA fragmentation caused by APAP hepatotoxicity (Fig. 4B). Therefore, A. muciniphila promoted the activation of the PI3K/Akt pathway, corrected the Bcl-2/Bax ratio, and further suppressed hepatic apoptosis.

### A. muciniphila enhanced gut barrier function and inhibited LPS stimulation.

**FIG 5:**
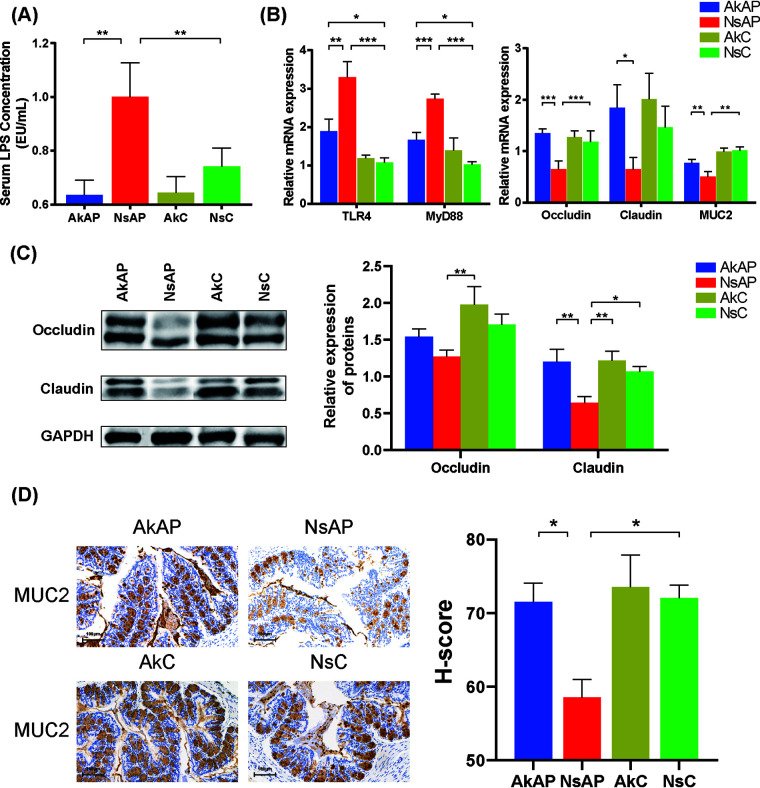
A. muciniphila enhanced gut barrier function. (A) Serum LPS concentration. (B) Relative hepatic and gut mRNA expression (liver, TLR4 and MyD88; gut, Occludin, Claudin, and MUC2). (C) Representative Western blot bands and quantitative analysis of protein expression. (D) Representative images of MUC2 immunohistochemical staining (original magnification × 40, scale bar = 100 μm) and H-score. The data are presented as the mean ± SEM. *, *P < *0.05; **, *P < *0.01; and ***, *P < *0.001 for the comparison. AkAP, A. muciniphila+APAP; AkC, A. muciniphila+saline control; NsAP, normal saline+APAP; NsC:=, normal saline+saline control; *n* = 6 each group for LPS detection; *n* = 10 each group for RT-qPCR; *n* = 6 each group for Western blot; *n* = 3 each group for immunohistochemical staining.

Disruption of gut barrier integrity is an extrahepatic characteristic of APAP intoxication (17). Our results demonstrated that serum lipopolysaccharide (LPS) levels were elevated in NsAP, and the elevation in LPS levels induced by APAP toxicity was significantly alleviated in AkAP (Fig. 5A). LPS is recognized by TLR4 in the liver and triggers MyD88-dependent inflammation. The mRNA expression of TLR4 and MyD88 in the liver was dramatically activated by APAP and effectively suppressed to normal levels by A. muciniphila, indicating restriction of APAP-mediated LPS leakage and bacterial translocation (Fig. 5B). Additionally, we assessed several typical gut barrier factors (Occludin, Claudin, MUC2, ZO-1, cannabinoid receptor 1, and cannabinoid receptor 2) in mRNA and protein expression levels (Fig. 5B and C and D). The mRNA expression of Occludin and MUC2 was downregulated by 45.8% and 50.3%, respectively, in NsAP compared with NsC, and A. muciniphila reinforced Occludin and MUC2 mRNA expression to a normal level. Significant difference of Occludin protein expression was only observed between NsAP and AkC. MUC2 protein expression followed a similar pattern to mRNA expression. The comparison between NsAP and NsC revealed that APAP significantly reduced Claudin protein level. And AkAP showed a recovered Claudin protein level compared with NsAP. APAP did not induce a reduction in Claudin mRNA expression, but A. muciniphila still exerted a stimulative effect. There was no significant difference in cannabinoid receptor 1, cannabinoid receptor 2, and ZO-1 levels among these groups (data not shown).

### A. muciniphila regulated APAP-induced intestinal dysbiosis.

**FIG 6:**
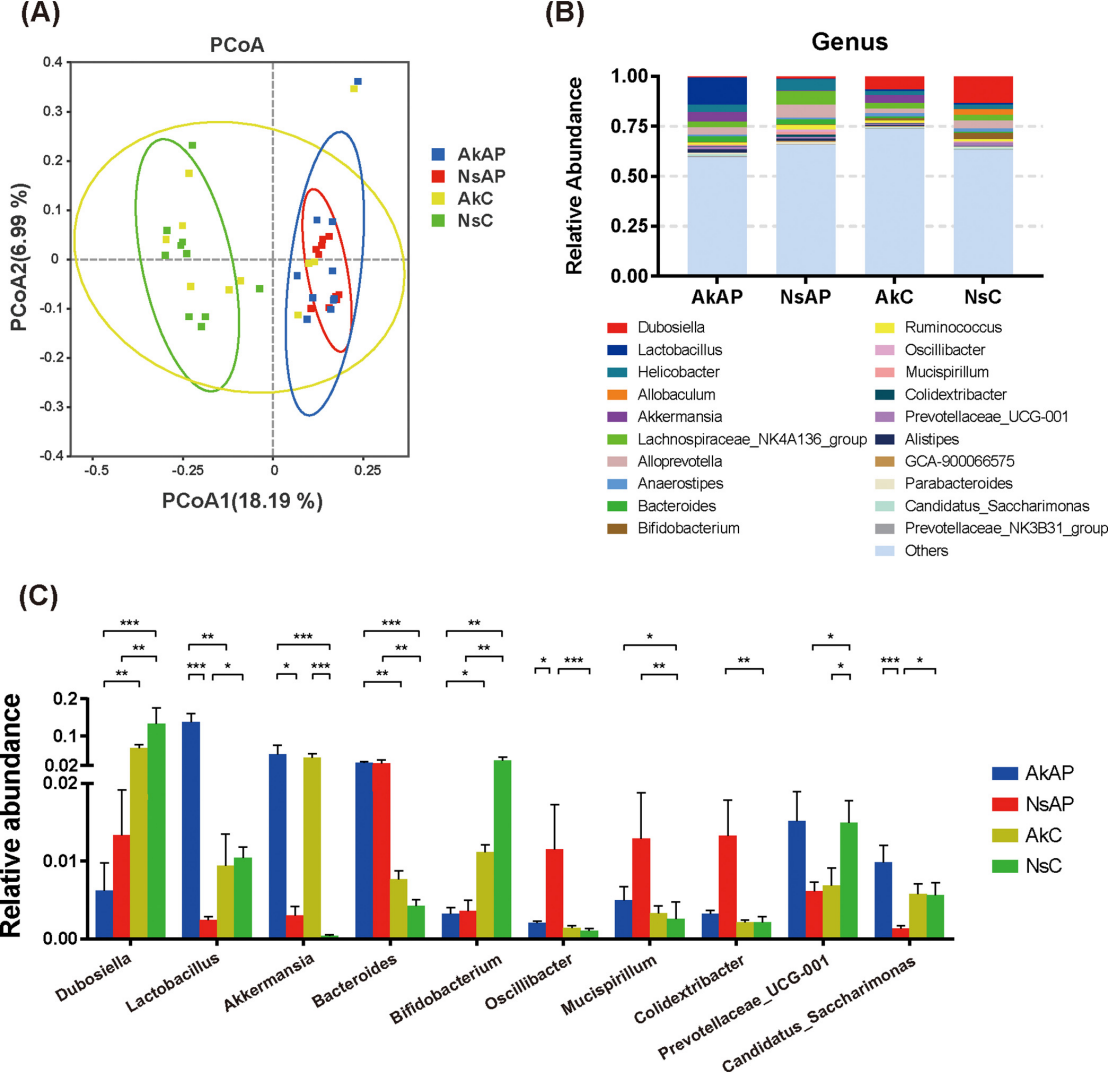
Modulatory effect of A. muciniphila on the alterations in microbial taxonomic abundance after APAP treatment. (A) Unweighted UniFrac PCoA plot. (B) Relative abundance of the 20 most abundant taxa at the genus level. (C) Bar chart of the relative abundance of genera with significant changes (Kruskal-Wallis test). The data are presented as the mean ± SEM. *, *P < *0.05; **, *P < *0.01; and ***, *P < *0.001 for the comparison. AkAP, A. muciniphila+APAP; AkC, A. muciniphila+saline control; NsAP, normal saline+APAP; NsC, normal saline+saline control; *n* = 10 each group.

To further reveal the extrahepatic influence of APAP overdose on intestinal homeostasis, we performed 16S rRNA sequencing analysis to explore the alteration in gut microbiota composition during APAP challenge and the influence of A. muciniphila administration on microflora structure. The Chao1 and Shannon index showed increased α-diversity after APAP treatment (Fig. S4).The fecal samples were generally clustered according to the similarity of the microflora and are illustrated by an unweighted UniFrac principal coordinates analysis (PCoA) plot (Fig. 6A). The microbiome profiles of AkAP and NsAP were distinctly separated from that of NsC. The taxonomic abundance of the microbiome was dramatically altered. The 10 most abundant phyla and 20 most abundant genera are presented in the Fig. S5 and Fig. 6B. At the phylum level, *Verrucomicrobioa* accumulated in AkAP and AkC upon gavage administration of A. muciniphila. Following APAP injection, the relative abundances of *Deferribacteres*, *Cyanobacteria*, and *Desulfobacterota* were considerably increased. The enrichment of *Actinobacteria* was significantly reduced after APAP treatment with or without *A. muciniphila* administration. The relative abundance of *Desulfobacterota* declined in AkAP compared with NsAP (Fig. S6). At the genus level, high proportions of *Bacteroides*, *Oscillibacter*, *Mucispirillum*, and *Colidextribacter* and decreased proportions of *Dubosiella*, *Lactobacillus*, *Bifidobacterium*, *Prevotellaceae_UCG-001* and *Candidatus_Saccharimonas* were observed in NsAP compared with NsC (Fig. 6C). *Lactobacillus* and *Candidatus_Saccharimonas* were markedly enriched, and *Oscillibacter* was markedly depleted, in AkAP compared with NsAP. Mice in AkAP showed a markedly higher abundance of *Lactobacillus* than mice in AkC. The abundance of *Candidatus_Saccharimonas* and *Oscillibacter* demonstrated no significant difference in AkAP compared with AkC or NsC. Next, we conducted linear discriminant analysis (LDA) effect size (LEfSe) analysis among AkAP, NsAP and NsC to confirm the alteration in the microbiota structure and identify the signature taxonomic markers (Fig. S7). According to the comparison of NsAP and NsC, *Oscillibacter*, *Mucispirillum*, *Colidextribacter*, and *Bacteroides* were enriched, and *Dubosiella*, *Bifidobacterium*, *Allobaculum*, *Prevotellaceae_UCG_001*, and *Lactobacillus* were depleted, by APAP treatment. Comparison between AkAP and NsAP showed that *Lactobacillus*, *Candidatus_Saccharimonas*, and *Akkermansia* were enriched in AkAP, while *Oscillibacter*, *Colidextribacter*, *Pseudaminobacter*, *Ruminiclostridium*, and *Idiomarina* were enriched in NsAP.

### The A. muciniphila-reshaped microbiota restored SCFA production.

**FIG 7:**
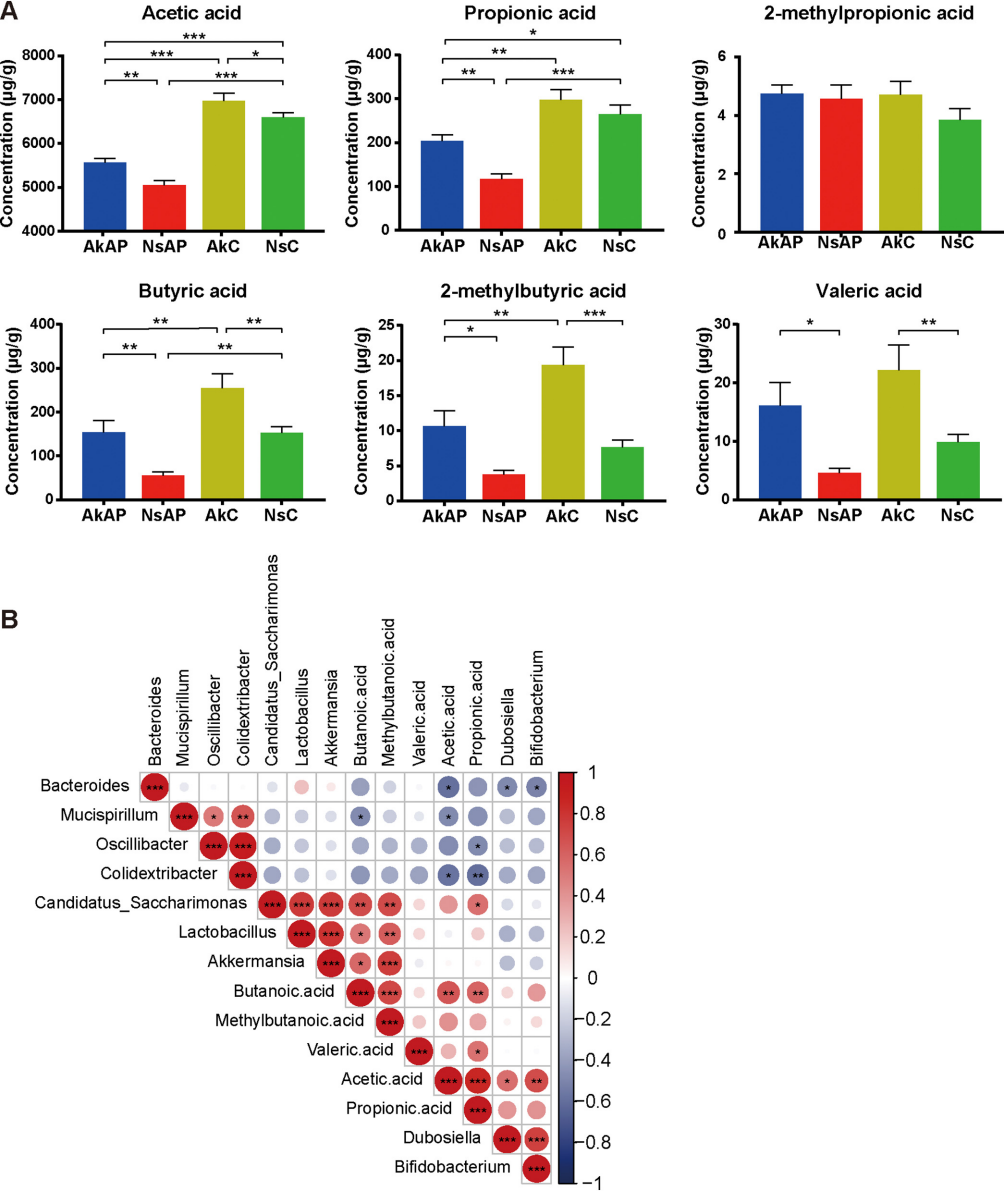
A. muciniphila improved SCFA production after APAP challenge. (A) Levels of SCFAs in fecal samples. (B) Correlation analysis of differentially abundant microbial genera and SCFAs. Correlation analysis was conducted by the Spearman rank correlation test, and red (positive correlation) and blue (negative correlation) colors represent different correlation coefficients. The data are presented as the mean ± SEM. *, *P < *0.05; **, *P < *0.01; and ***, *P < *0.001 for the comparison. AkAP, A. muciniphila+APAP; AkC, A. muciniphila+saline control; NsAP, normal saline+APAP; NsC, normal saline+saline control; *n* = 10 for each group.

SCFAs, which are only produced by microbial fermentation in hosts, are regarded as indispensable signals in the liver-gut axis. Therefore, we conducted a targeted metabolomic analysis to determine the concentrations of specific SCFAs (acetic acid, propionic acid, 2-methylpropionic acid, butyric acid, 2-methylbutyric acid, and valeric acid) in feces (Fig. 7A). AkC presented consistent enhancement in the levels of acetic acid, butyric acid and 2-methylbutyric acid compared with NsC, indicating that A. muciniphila elicits SCFA production. The concentrations of acetic acid, propionic acid and butyric acid were strikingly decreased by APAP injection with or without A. muciniphila administration, demonstrating that APAP suddenly reduced SCFA production. A. muciniphila increased the levels of these SCFAs, although the restored levels remained lower than those in NsC and AkC. APAP-treated mice showed elevation in 2-methylbutyric acid and valeric acid elevations, but the difference was not significant. Even so, A. muciniphila still increased the concentrations of these SCFAs after APAP injection to conventional levels. No intergroup variation was observed for 2-methylpropionic acid.

### Correlations of differentially abundant microbial genera and representative biomarkers.

Furthermore, we performed correlation analysis involving differential genera, SCFAs and injury indicators to determine the putative association between altered intestinal genera and biomarkers. The correlation analysis of differentially abundant microbial genera and SCFAs showed that *Lactobacillus* and *Candidatus_Saccharimonas* were conserved under *Akkermansia* administration, as their relative abundances were positively correlated with that of *Akkermansia.* Corresponding positive correlations were identified among *Oscillibacter*, *Mucispirillum*, and *Colidextribacter*, as these genera were regarded as unfavorable genera and were enriched in NsAP. Acetic acid was positively correlated with *Dubosiella* and *Bifidobacterium* and negatively correlated with *Bacteroides*, *Mucispirillum*, and *Colidextribacter.* Propionic acid was positively correlated with *Candidatus_Saccharimonas* and negatively correlated with *Oscillibacter* and *Colidextribacter.* Butyric acid and 2-methylbutyric acid were positively correlated with *Akkermansia*, *Lactobacillus*, and *Candidatus_Saccharimonas* (Fig. 7B).

**FIG 8:**
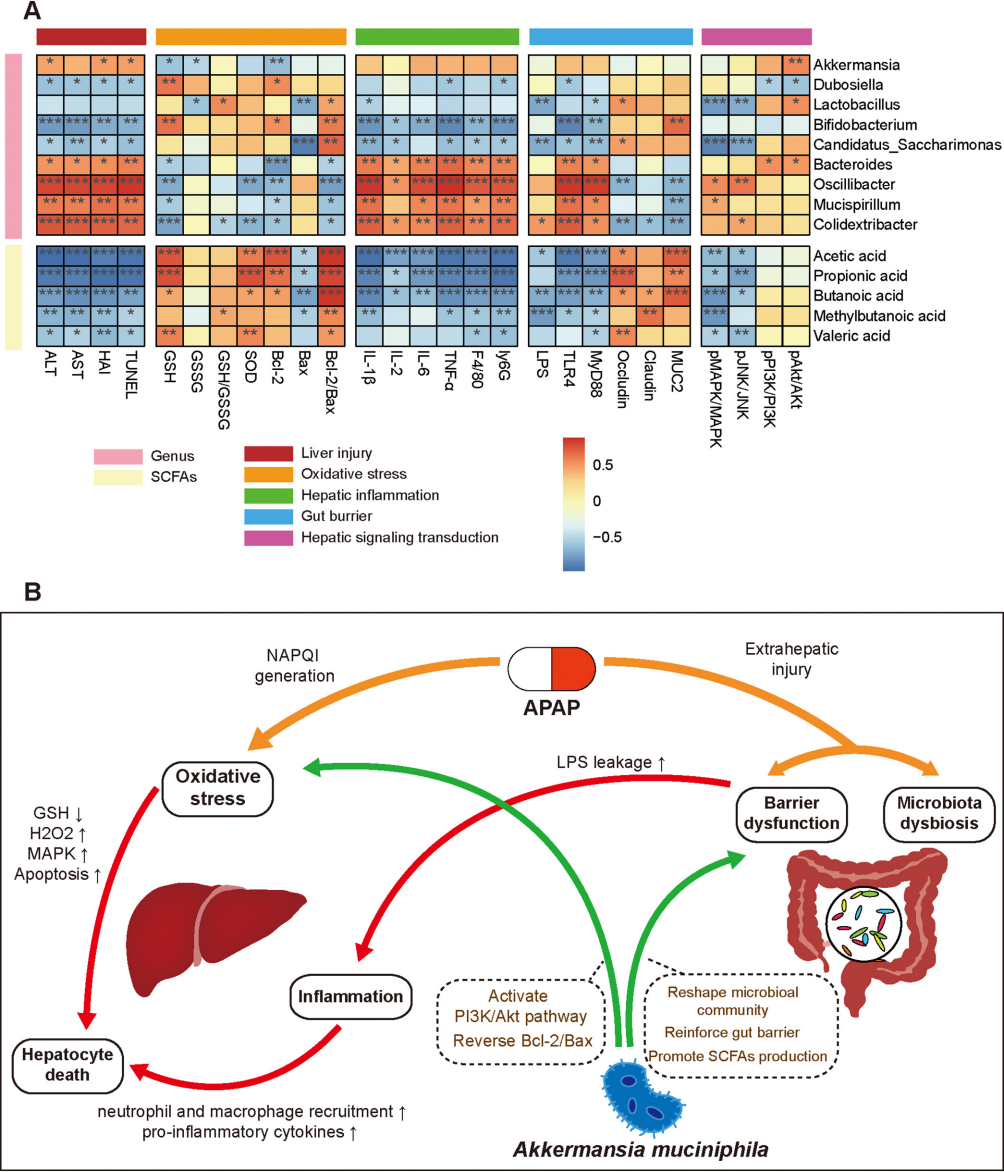
The interactions among the gut microbiota, microbial metabolism, and APAP-induced pathological changes. (A) Correlation heatmap of differentially abundant microbial genera, SCFAs, and representative biomarkers. Red (positive correlation) and blue (negative correlation) colors represent different correlation coefficients. *, *P < *0.05; **, *P < *0.01; and ***, *P < *0.001. (B) Schematic diagram of A. muciniphila intervention in APAP-induced liver injury.

Dysbiotic bacteria might aggravate the severity of APAP-induced liver injury. *Oscillibacter*, *Mucispirillum*, and *Colidextribacter*, which were abundant in NsAP, had positive associations with the liver injury indices (ALT, AST, HAI, and TUNEL positivity rate) and inflammatory factors (cytokines and inflammatory cell infiltration). In contrast, *Lactobacillus*, *Candidatus_Saccharimonas*, and SCFAs were generally negatively associated with the liver injury indices, inflammatory factors, serum LPS level, and JNK phosphorylation. In addition, the antioxidant capacity (Bcl-2/Bax ratio and SOD activity) was positively correlated with *Lactobacillus*, *Candidatus_Saccharimonas*, *Bifidobacterium* and SCFAs. The levels of Akt phosphorylation were positively correlated with *Akkermansia*, *Lactobacillus*, and *Bacteroides* (Fig. 8A). Based on the integrated analysis, we revealed the essential interaction among the A. muciniphila*-*reshaped microbial community, microbial metabolic function and hepatoprotective effect of A. muciniphila.

## DISCUSSION

Bidirectional interaction between the gut microbiota and liver, which is termed the gut–liver axis, is accomplished in multiple ways, such as bile acid circulation, LPS leakage, and transmission of inflammatory mediators (18). Individualized microbial composition is considered a potential factor underlying the variable specificity of drug metabolism in the liver. It has been reported that the intestinal microflora contributes to susceptibility to APAP-induced hepatoxicity (9). This may be due to competitive inhibition against APAP by gut-derived metabolites or LPS-induced augmentation of hepatic inflammation, but the exact mechanism remains unclear (17). Probiotics have been regarded as a therapeutic strategy for liver diseases and the concomitant gut dysbiosis. The beneficial activities of A. muciniphila in improving immune and metabolic disorders have been well recognized in liver disease, IBD, and type 2 diabetes (14, 19). However, recent studies suggest that A. muciniphila aggravates acute graft-versus-host disease (aGVHD) and contributes to enhance pathogen susceptibility of Citrobacter rodentium and Clostridium difficile (20, 21). Therefore, it’s necessary to investigate the effects of A. muciniphila in specific conditions. Our study revealed that A. muciniphila administration notably attenuated APAP-induced hepatocellular death by restoring the GSH/GSSG equilibrium, inhibiting mitogen-activated protein kinase (MAPK) activation, and alleviating oxidative stress and inflammation in the liver. Its beneficial effect was accompanied by activation of the PI3K/Akt pathway and mediated by regulation of the composition and metabolic function of the intestinal microbiota (Fig. 8B).

Following the undesired formation of NAPQI, mitochondrial oxidative stress and dysfunction are considered the primary pathogenic mechanisms of APAP hepatotoxicity. GSH/GSSG imbalance and diminished SOD activity indicate severe disruption of the antioxidant system against superoxide radicals (2). The accumulation of reactive species that are generated by the electron transport chain and NADPH oxidases and not scavenged immediately leads to lipid peroxidation and DNA oxidative damage (22). A number of studies have found that APAP treatment promotes the generation of myeloperoxidase (MPO) and malondialdehyde (MDA), depletes GSH and reduces SOD activity, consequently compromising the buffering capacity against oxidative stress events in the liver (23, 24). Consistent with these studies, our results showed a decreased GSH/GSSG ratio and SOD activity under APAP intoxication, and A. muciniphila administration restored the antioxidant levels, suggesting that A. muciniphila corrected the redox imbalance. Because A. muciniphila mitigates oxidative stress in hepatocytes, the activation of the downstream MAPK pathway was further suppressed. This was supported by our Western blot analysis, which showed that A. muciniphila inhibited JNK and ERK1/2 phosphorylation. JNK and ERK MAPKs are vital intracellular signaling pathways that are responsible for apoptosis, inflammation, and proliferation (25). Activation of MAPKs is a necessary condition for APAP-induced hepatocyte death due to its amplification of mitochondrial oxidative stress and downstream transduction of the MAPK signaling pathway (26, 27). Previous studies have indicated that JNK inhibitors or ERK1/2 inhibitors protect against APAP hepatotoxicity (28, 29).

In addition to oxidative stress, inflammation is recognized as a necessary aggravated cofactor during the progression of APAP hepatotoxicity (30). The levels of proinflammatory cytokines such as IL-1β and TNF-α generally increase in APAP-induced liver injury (31, 32). Kupffer cells are continuously stimulated by gut-derived bacterial components such as LPS, inducing low-grade inflammation. The TLR4 receptor on the Kupffer cell membrane binds to LPS and triggers inflammation via MyD88-dependent signaling (33). Increased intestinal permeability allows vast translocation of bacterial components into the portal vein, leading to portal endotoxemia and accelerating liver disease progression (15, 34). As an agent causing extrahepatic damage, overdose of APAP destroys the gut barrier and enhances bacterial translocation, exacerbating hepatic inflammation (17, 35). Inhibition of LPS-binding protein (LBP) effectively reduces intrahepatic cytokine production and susceptibility to APAP-induced liver injury (36). In this study, A. muciniphila suppressed hepatic inflammation, as evidenced by the reduced expression of IL-1β and TNF-α and reduced infiltration of macrophages and neutrophils. Furthermore, we found that A. muciniphila reduced serum LPS levels, downregulated hepatic TLR4 and MyD88 expression, and upregulated intestinal Occludin and MUC2 expression as well as SCFA production, indicating that its anti-inflammatory property was closely associated with reinforcement of APAP-impaired gut barrier function.

Hepatocyte death caused by APAP hepatotoxicity is a complex process mediated by the coordination of multiple intracellular signal transduction pathways (1, 27). A variety of apoptosis-related pathways, such as the AMPK-dependent pathway and SIRT1 pathway, are known to participate in this process. Our transcriptomic results showed that modulation of the PI3K/Akt pathway is a considerable hepatoprotective mechanism of A. muciniphila. Moreover, we examined Akt phosphorylation and found that it was improved after A. muciniphila administration. The PI3K/Akt pathway plays an important role in liver injury and regeneration. Recent studies have revealed that the PI3K/Akt pathway alleviates liver injury by phosphorylating target proteins such as Bcl-2, NF-κB and Nrf2 (37). Bcl-2 family proteins are the classic downstream regulators of activated Akt and further regulate DNA repair and apoptosis (38). The Bcl-2 family of proteins contains both pro- and antiapoptotic members and governs apoptotic mitochondrial events. The proportion of proapoptotic proteins and antiapoptotic proteins determines cell persistence to the apoptotic response (39). Activated Akt can enhance the antiapoptotic protein Bcl-2 and inhibit the proapoptotic protein Bax, thus alleviating apoptosis and promoting cell survival (16, 40). Consistent with these findings, our experiments showed that A. muciniphila increased the Bcl-2/Bax ratio, accompanied by reduced TUNEL positivity, suggesting that A. muciniphila alleviated apoptosis by regulating Bcl-2 family proteins via the PI3K/Akt pathway (41).

Recent studies have increasingly focused on the interaction mechanism of the liver-gut axis and the application of probiotics in liver disease (42, 43). Intestinal dysbiosis has been demonstrated as a latent factor involved in APAP-induced liver injury, as germfree animals present a lower susceptibility to APAP hepatotoxicity (44). Modulation of other probiotic strains, such as Lactobacillus acidophilus LA14 and Lactobacillus rhamnosus GG, protects against APAP-induced liver injury (44, 45). In our study, we found that opportunistic pathogens, such as *Oscillibacter*, *Colidextribacter*, and *Mucispirillum*, were highly enriched in NsAP, and the abundances of these genera were positively correlated with the liver injury indices, barrier disruption, and hepatic inflammation. *Oscillibacter* shows a close association with depression and obesity. Previous studies found enrichment of the genus *Oscillibacter* in patients with chronic metabolic disorders such as obesity and NAFLD (46, 47). APAP increased the level of *Mucispirillum*, which is linked to IBD and regarded as an aggravated factor associated with intestinal inflammation (48, 49). Potential probiotic genera, such as *Dubosiella*, *Lactobacillus*, *Bifidobacterium*, and *Candidatus_Saccharimonas* were depleted by APAP treatment, whereas the abundances of *Lactobacillus* and *Candidatus_Saccharimonas* were observably reversed by A. muciniphila administration (50, 51). Many studies have demonstrated that *Lactobacillus* can stimulate Treg cell proliferation, promote SCFA production and prevent epithelial barrier dysfunction, exerting immunomodulatory effects (52, 53). Importantly, the abundance of *Lactobacillus* is decreased in a variety of liver diseases, and supplementation with *Lactobacillus* shows a protective effect against liver damage (44, 45, 54, 55). Previous studies have reported the protective effect of *Lactobacillus* administration against APAP-induced liver injury via modulation of BclII/Bax, activation of the Nrf-2 antioxidant pathway and induction of autophagy (44). In our study, we found that the abundance of *Lactobacillus* was positively correlated with Akt phosphorylation, identifying that *Lactobacillus* played a pivotal role in the PI3K/Akt-mediated antioxidant response. Activation of the PI3K/Akt pathway by *Lactobacillus* strains has been realized in other studies, although the underlying mechanism still needs further investigation.

As the APAP-treated mice showed the disruption of microbial homeostasis, we hypothesized that microbiota-derived metabolites might be key mediators of the response to APAP-induced pathological conditions. SCFAs are metabolic products generated by the cecal and colonic microbiota by fermenting nondigestible dietary fibers (56). The results of targeted metabolomics revealed that fecal SCFA concentrations were considerably reduced by the impact of APAP due to the depletion of SCFA-producing commensals, such as *Lactobacillus* and *Bifidobacterium*. A. muciniphila is an SCFA-producing bacterial strain itself (57). In addition, the A. muciniphila*-*reshaped microbial community was dominated by beneficial bacteria in favor of SCFA synthesis. In this context, A. muciniphila restored acetate, propionate and butyrate to appropriate levels, as expected. Propionate and butyrate were negatively correlated with gut barrier dysfunction, suggesting that SCFAs reinforced the mucosal barrier under the extrahepatic impact of APAP. Butyrate is absorbed by colonic epithelial cells as an energy source, thereby supporting epithelial cell proliferation and maintaining gut barrier integrity (58, 59). Increased butyrate levels suppress intestinal inflammation and chemotaxis by stimulating the differentiation of Treg cells via GPR signaling activation or histone deacetylase (HDAC) inhibition (60–62). SCFAs can also be transported into the liver by the portal vein and play an important role in hepatic pathological progression (63, 64). Butyrate-producing strains alleviate steatohepatitis and autoimmune hepatitis through enterohepatic immunologic modulation of T cell differentiation (65). In addition, as an HDAC inhibitor, SCFAs probably suppress oxidative stress during APAP-induced mitochondrial dysfunction (66).

Taken together, the results of our study demonstrated that A. muciniphila protects against APAP-induced hepatotoxicity by ameliorating oxidative stress and inflammation. With latent risk, the disrupted gut barrier is partly recovered. The hepatoprotective effect of A. muciniphila was closely correlated with the reshaped gut microbiota and reinforced SCFA secretion.

## MATERIALS AND METHODS

### Preparation of A. muciniphila strains.

A. muciniphila Muc^T^ was purchased from ATCC (ATCC Number: BAA-835) and cultured in 0.5% (wt/vol) mucin-supplemented brain heart infusion (BHI) (Difco, MI, USA) medium anaerobically at 37°C. After 48 h of incubation, the cultures were centrifuged at 7,000 g for 15 min, washed twice in sterile saline, and resuspended at a bacterial concentration of 1.5 × 10^10^ CFU/mL by measuring absorbance at 630 nm for further intragastric administration.

### Animals and treatment.

Specific pathogen-free (SPF) C57BL/6 mice (male, 6 weeks) were purchased from Shanghai SLAC Laboratory Animal Co., Ltd., and maintained in an SPF environment. After 2 weeks of adaptive feeding, the mice were randomly assigned into four groups (*n* = 10 each group) (Fig. 1A). The mice in the groups AkAP (A. muciniphila+APAP) and AkC (A. muciniphila+saline control) were orally gavaged with 3 × 10^9^ CFU *A. muciniphila* suspended in 0.2 mL of sterile saline per day, while those in the groups NsAP (normal saline+APAP) and NsC (normal saline+saline control) were treated with an equivalent dose of sterile saline. The treatments were administered for 14 days. After 14 days of gavage treatment, a model of APAP-induced liver injury was established at day 15 as previously described (9). Briefly, following 12 h of fasting, mice in the groups AkAP and NsAP were injected intraperitoneally with APAP (300 mg/kg in normal saline), while those in AkC and NsC were administered an equivalent volume of normal saline as a control. Fresh feces were collected 24 h after intraperitoneal injection, followed by sacrifice. Blood, liver tissues, colon tissues, and colon contents were harvested for further experiments. All mice were housed at ambient temperature under humidity-controlled conditions with an automatic 12 h light-dark cycle and provided with chow diet and water *ad libitum*. All experiments were approved by the Animal Care and Use Committee of the First Affiliated Hospital, School of Medicine, Zhejiang University.

### Serum and liver biochemical parameter analysis.

Serum ALT and AST levels were evaluated by a dry chemistry analyzer (FUJI DRI‐CHEM 7000V, FUJIFILM, Tokyo, Japan).

Liver GSH and GSSG levels were measured by a green fluorescence assay kit (ab205811, Abcam, Cambridge, UK), Liver SOD activity was measured by a colorimetric assay kit (Sigma–Aldrich, MO, USA), and the serum LPS level was measured by a limulus amebocyte lysate (LAL) chromogenic endpoint assay kit (Hycult Biotech, Uden, the Netherlands) according to the manufacturer’s instructions.

### Histology and immunohistochemistry.

The left central liver lobe and proximal colon was freshly isolated, fixed with 10% formalin for 24 h, embedded in paraffin. Liver sections were stained with H&E for pathological assessment. Three independent visual fields were randomly picked at ×20 magnification in each specimen. Liver necrosis and inflammation were evaluated according to the modified Knodell HAI by a pathologist who was blinded to this study (67).

Immunohistochemical analysis was performed using standard procedures (68). Paraffin-embedded sections were deparaffinized and rehydrated. Subsequently, liver sections were incubated with anti-F4/80 (Cell Signaling Technology, MA, USA) or anti-Ly6G (Abcam) antibodies to estimate the infiltration of macrophages and neutrophils, and colon sections were incubated with anti-Mucin 2 (MUC2, Abcam) or anti-zonula occludens-1 (ZO-1, Proteintech) to estimate gut barrier proteins. Five independent visual fields were randomly picked at ×40 magnification in each specimen. The relative quantification of staining intensity was performed by calculating the H-score by using ImageJ software. The formula for the H-score was as follows: H-score = low positive staining percentage × 1 + positive staining percentage × 2 + high positive staining percentage × 3 (69).

### Flow cytometry.

Fresh livers were isolated and dissociated using gentleMACS Dissociator (Miltenyi Biotec, Bergisch Gladbach, Germany) and Mouse Liver Dissociation Kit (Miltenyi) according to the manufacturer’s protocols. The cell suspension was filtered through a 70 μm cell strainer, followed by Percoll density gradient centrifugation to enrich leukocytes. The antibodies used in cytometry are listed in Table S2. Flow cytometry analysis was performed on CytoFLEX LX (Beckman Coulter, CA, USA).

### TUNEL assay.

Apoptosis of hepatocytes was evaluated by employing the TUNEL Apoptosis Detection Kit (Vazyme, Nanjing, China) according to the manufacturer’s instructions. Briefly, paraffin-embedded liver sections were deparaffinized, rehydrated, and blocked, followed by incubation with the TUNEL labeling solution for 2 h at room temperature. Thereafter, the sections were immediately observed and imaged using a fluorescence microscope. Five independent visual fields were randomly picked at ×20 magnification in each specimen. TUNEL-positive cells were counted by ImageJ software.

### 16S rRNA gene sequencing.

The feces collected before sacrifice were used for 16S rRNA sequencing. Fecal genomic DNA was extracted with the Qiagen Power PowerSoil Kit (Qiagen, Hilden, Germany) according to the manufacturer’s protocols. The V3-V4 variable region of 16S rRNA were amplified by universal primers (Table S1). The amplicon was used to construct sequencing libraries after purification, followed by sequencing performed on the Illumina NovaSeq platform. Paired‐end reads were generated and filtered through Trimmomatic software. Qualified reads were merged, denoised, and clustered to generate operational taxonomic units (OTUs) by using QIIME software and Vsearch software. Subsequently, the Silva database was employed to annotate OTUs. α-Diversity was determined with the Shannon and Chao1 diversity indices. PCoA and LEfSe analysis were conducted to visualize β-diversity by using R software.

### Targeted fecal metabolomics.

Targeted metabolomics was carried out using gas chromatography-mass spectrometry (GC-MS) to quantify the levels of representative SCFAs. Feces were suspended in ultrapure water, homogenized, centrifuged, and filtered. The filtrate was blended with an equal volume of ethyl acetate and incubated at 4°C for 30 min, followed by centrifugation to separate and collect the ethyl acetate layer. Different concentrations of SCFA standards were treated with the same procedures. SCFAs in the samples were characterized through the comparison of their retention times with those of standards. Calibration curves were established based on the peak areas of the standards at different concentrations to quantify SCFA levels.

### RNA extraction.

Total RNA from stored liver segments was extracted using the Qiagen RNeasy Plus mini kit (Qiagen) according to the instructions provided. The quality and quantity of RNA were measured by a NanoDrop 2000 spectrophotometer (Thermo Fisher, MA, USA), and the RNA integrity number (RIN) was determined by an Agilent 2100 Bioanalyzer (Agilent Technologies, CA, USA). Only RNAs satisfying the criteria (RNA concentration ≥ 2,500 ng/μL and RIN ≥ 7) were subjected to RNA sequencing or quantitative real-time PCR (RT-qPCR).

### Transcriptome sequencing.

Four micrograms of the total RNA collected above were purified with Agencourt AMPure XP (Beckman Coulter, CA, USA) magnetic beads to enrich the mRNA. Then, the mRNA was randomly fragmented into small pieces and subjected to template construction of the mRNA library with the TruSeq Stranded mRNA LT Sample Prep Kit (Illumina, CA, USA). Then, 150-bp paired-end reads were generated through sequencing conducted on the Illumina HiSeq 2500 platform (Illumina). After filtration using Trimmomatic, trimmed reads were mapped to the reference genome using Hisat2. DEGs were identified by the DESeq2 package in R, and these DEGs were aligned against the KEGG database for pathway enrichment analysis.

### RT-qPCR.

To validate the transcriptomic results and detect target gene expression precisely, RT-qPCR was applied to quantify mRNA levels. Briefly, 500 ng of RNA was subjected to reverse transcription using PrimeScript RT Master Mix (TaKaRa Biomedicals, Kusatsu, Japan), and subsequently, the synthesized cDNA was used for RT-qPCR with TB Green Premix *Ex Taq* II (TaKaRa) and paired primers on a ViiA real-time PCR system (Applied Biosystems, CA, USA). The reaction of each sample was performed in duplicate.

The relative expression of target genes was standardized to the GAPDH mRNA levels, and fold changes between groups were calculated based on the 2^-△△Ct^ method. All paired primer sequences are listed in Table S1.

### Western blotting.

Liver samples (30 mg) were added to 1 mL of radioimmunoprecipitation (RIPA) lysis buffer containing 1% protease inhibitor and 1% phosphatase inhibitor, fully homogenized for 30 min and centrifuged at 10,000 × *g* for 15 min. The supernatants were diluted to the same protein concentration and mixed with NuPage loading buffer (Invitrogen, CA, USA) prior to being denatured by boiling for 5 min. Proteins were electrophoresed, transferred onto polyvinylidene fluoride (PVDF) membranes, and incubated with primary antibodies and secondary antibodies successively. Bands were visualized with ECL detection reagents (Beyotime, Beijing, China) and captured. Protein expression was quantified in an optical densitometric manner using ImageJ software. All antibodies used are listed in Table S2.

### Statistical analysis.

Most data were analyzed by one-way analysis of variance (ANOVA) with Fisher’s LSD *post hoc* test conducted using SPSS 20.0 (SPSS, Inc., IL, USA) and are presented as the means ± standard deviations (SDs). A *P value* < 0.05 indicated statistical significance. Graphs were plotted using GraphPad Prism 7.0 (GraphPad Software Inc., CA, USA) and R software.

### Data availability.

The 16S rRNA gene sequencing data are available at NCBI’s Sequence Read Archive (SRA) database under the BioProject accession code PRJNA750743. The raw data of RNA sequencing are available at SRA database under the BioProject accession code PRJNA751139.
