# Supplementary material for: Akkermansia muciniphila Ameliorates Acetaminophen-Induced Liver Injury by Regulating Gut Microbial Composition and Metabolism
Source: Microbiol Spectr. 2022 Feb 2;10(1):e01596-21. doi: 10.1128/spectrum.01596-21 (PMC8809353; doi:10.1128/spectrum.01596-21)

Supplementary materials

Akkermansia muciniphila **ameliorates acetaminophen-induced liver injury by regulating gut microbial composition and metabolism**

Supplementary Table S1 Primer pairs of Real-time Quantitative PCR analysis and 16S rRNA sequencing.

Gene name	Forward primer sequence (5'-3')	Reverse primer sequence (5'-3')
IL-1β	GCCACCTTTTGACAGTGATGAG	ATGTGCTGCTGCGAGATTTG
IL-2	GAAACTCCCCAGGATGCTCA	CGCAGAGGTCCAAGTTCATCT
IL-6	GAGGATACCACTCCCAACAGACC	AAGTGCATCATCGTTGTTCATACA
IL-10	AGGCGCTGTCATCGATTTCT	ATGGCCTTGTAGACACCTTGG
TNF-α	GCGCCAAGCATTCAATGAGC	ATCTCTTCCCCACCCCGAAT
IFN-γ	GAGGTCAACAACCCACAGGT	GGGACAATCTCTTCCCCACC
TLR4	CACCAGGAAGCTTGAATCCCT	GGAATGTCATCAGGGACTTTGC
MyD88	GCCAGATTCTCTGATGCCGT	TGGGAGGAAAGGCAGTCCTA
Occludin	TTCCTCTGACCTTGAGTGTGG	CTCTTGCCCTTTCCTGCTTT
Claudin	GCCATCTACGAGGGACTGTG	CCCCAGCAGGATGCCAATTA
MUC2	GAAGCCAGATCCCGAAACCA	GAATCGGTAGACATCGCCGT
GAPDH	TGCGACTTCAACAGCAACTC	ATGTAGGCAATGAGGTCCAC

16S rRNA universal primer	Forward primer sequence (5'-3')	Reverse primer sequence (5'-3')
Bacteria16S rRNA V3V4	TACGGRAGGCAGCAG	AGGGTATCTAATCCT

Supplementary Table S2. List of the antibodies we used.

Antibody name	Catalog number	Manufacturer
Phospho-SAPK/JNK (Thr183/Tyr185) Rabbit mAb	4668T	Cell Signaling Technology, MA, USA
SAPK/JNK Antibody	9252T	Cell Signaling Technology
Phospho-SAPK/JNK (Thr183/Tyr185) Rabbit mAb	4668T	Cell Signaling Technology
SAPK/JNK Antibody	9252T	Cell Signaling Technology
Phospho-NF-κB p65 (Ser536) Rabbit mAb	3033S	Cell Signaling Technology
NF-κB p65 Rabbit mAb	8242S	Cell Signaling Technology
Phospho-PI3 Kinase p85 (Tyr458)/p55 (Tyr199) Rabbit mAb	17366S	Cell Signaling Technology
PI3 Kinase p85 Rabbit mAb	4257S	Cell Signaling Technology
Phospho-Akt (Ser473) Rabbit mAb	4060T	Cell Signaling Technology
Akt (pan) Rabbit mAb	4691T	Cell Signaling Technology
Anti-Bcl-2 antibody	ab182858	Abcam, Cambridge, UK
Anti-Bax antibody	ab32503	Abcam
F4/80 Rabbit mAb	70076S	Cell Signaling Technology
Anti-Ly6g antibody	ab238132	Abcam
GAPDH Rabbit mAb	5174S	Cell Signaling Technology
Goat Anti-Rabbit IgG H&L (HRP)	ab6721	Abcam
Ms CD45 APC-Cy7 30-F11	557659	BD Pharmingen, CA, USA
Ms CD3e PE-Cy7 145-2C11	552774	BD Pharmingen
Ms CD4 PerCP-Cy5.5 RM4-5	550954	BD Pharmingen

Antibody name	Catalog number	Manufacturer
Ms CD8a FITC 53-6.7	553030	BD Pharmingen
Ms NK-1.1 PE PK136	557391	BD Pharmingen
Ms CD45R/B220 APC RA3-6B2	553092	BD Pharmingen
CD11b FITC M1/70	557396	BD Pharmingen
Ms F4/80 PE T45-2342	565410	BD Pharmingen
Ms CD86 PE-Cy7 GL1	560582	BD Pharmingen
Ms LY-6G PE-CF594 1A8	562700	BD Pharmingen
Ms CD11c APC HL3	550261	BD Pharmingen
Ms I-A/I-E BB700 2G9	746086	BD Pharmingen
Ms CD16/CD32 Pure 2.4G2	553141	BD Pharmingen
Anti-ZO-1 antibody	21773-1-AP	Proteintech
Anti-MUC2 antibody	ab272692	Abcam

Supplementary Table S3. List of all hub genes in PPI network of AkAP vs. NsAP.

Hub gene in cluster 1	Log2(fold change) in transcriptome (AkAP vs NsAP)	P-value	Regulation
Cyp1a2	1.768216897	1.86E-06	Up
Cyp26a1	2.594326706	7.97E-09	Up
Cyp26b1	2.318046222	0.012303076	Up
Cyp2b9	2.850040688	1.43E-13	Up
Cyp2c29	2.042037026	0.001020283	Up
Cyp2c37	2.545121855	1.29E-05	Up
Cyp2c38	3.158431295	8.78E-14	Up
Cyp2c39	1.651934748	4.06E-06	Up
Cyp2c40	1.976307396	2.50E-06	Up
Cyp2c50	1.638424284	6.98E-05	Up
Cyp2c54	1.480935742	0.000360336	Up
Cyp2c55	2.639843946	2.54E-07	Up
Cyp2d11	2.035431295	0.002195417	Up
Cyp2e1	1.820333662	1.17E-05	Up
Cyp3a11	1.880891784	2.59E-06	Up
Cyp3a25	1.660749721	7.38E-06	Up
Cyp3a44	-1.840833924	0.038991957	Down
Cyp4a12a	1.684305795	0.002123091	Up
Cyp4a12b	1.85927405	0.001629621	Up
Cyp4a31	-1.16465262	0.000187946	Down
Cyp7a1	4.396771978	3.78E-12	Up
Gsta1	-1.492051705	4.29E-09	Down
Gsta3	1.085498672	0.010769894	Up
Gstm2	1.538534664	0.000316236	Up
Gstm3	2.018723984	8.59E-07	Up
Gstm6	1.515935433	0.000158622	Up
Gstm7	1.211866304	1.74E-06	Up
Gstt3	2.136960968	1.02E-05	Up
Hsd3b2	2.195647846	5.87E-05	Up
Hsd3b5	3.462906202	2.15E-05	Up
Ugt1a1	1.151061134	6.54E-07	Up
Ugt1a5	2.338004394	7.60E-06	Up
Ugt2a3	1.729989381	0.000404776	Up
Ugt2b1	2.234934708	0.000388228	Up
Ugt2b36	1.073512007	0.002802096	Up
Ugt2b38	1.329915086	0.000127909	Up

Hub gene in cluster 2	Log2(fold change) in transcriptome (AkAP vs NsAP)	P-value	Regulation
Bdkrb2	-1.769639042	0.012605161	Down
C5ar1	-2.152098967	0.000137104	Down
Ccl3	-1.709122052	0.043748195	Down
Ccr1	-2.456539669	0.000576772	Down
Ccr2	-1.335944056	0.019267499	Down
Cd14	-2.938243721	0.005184392	Down
Cx3cl1	-1.286216372	0.00517584	Down
Cx3cr1	-1.447727877	0.021922354	Down
Cxcl1	-4.06510802	1.9646E-09	Down
Cxcl10	-1.331227121	0.031996649	Down
Cxcl2	-2.082708759	0.002035576	Down
Cxcr2	-3.220418122	7.8105E-06	Down
Cxcr4	-2.777107248	5.39551E-07	Down
Gngt1	1.63644586	0.002265499	Up
Icam1	-1.360223319	2.61635E-10	Down
Il11	-2.133284284	0.016907752	Down
Il15	1.090872059	0.013070902	Up
Il18	1.104417016	0.000197179	Up
Il1b	-1.733607912	0.008769316	Down
Il1rn	-1.871261678	0.000144364	Down
Il7	-1.165219192	0.040729658	Down
Socs3	-2.644763901	1.79802E-32	Down

Hub gene in cluster 3	Log2(fold change) in transcriptome (AkAP vs NsAP)	P-value	Regulation
Cps1	1.232026315	8.20205E-05	Up
Fga	-1.226682331	6.21485E-10	Down
Fgb	-1.259025096	1.80589E-10	Down
Fgg	-1.098064104	2.55763E-08	Down
Hpd	1.724896952	2.05123E-05	Up
Mat1a	1.294367448	0.001634125	Up
Otc	1.276484782	0.001906147	Up
Pah	1.823060696	0.000703115	Up
Rgn	1.261799194	0.000287457	Up
Slc27a5	1.027489552	4.00928E-05	Up
Slco1b2	1.720874773	0.000330299	Up
Tdo2	1.117265191	0.020057658	Up

Hub gene in cluster 4	Log2(fold change) in transcriptome (AkAP vs NsAP)	P-value	Regulation
Aldh4a1	1.22378612	3.78E-07	Up
Arg2	-2.284224115	0.003191701	Down
Ccl2	-1.679271723	0.030013972	Down
Col4a1	-1.248065513	0.020427421	Down
Col4a4	2.555087458	0.002278573	Up
Col6a1	1.091645922	0.000143106	Up
Col6a2	1.215577961	3.96E-05	Up
Col6a6	-3.096716551	2.54E-11	Down
Fos	-3.805369462	0.000328761	Down
Il1r1	-1.779604443	0.001090089	Down
Il1r2	-3.039552534	0.044115462	Down
Itga2	-2.855649929	0.000240522	Down
Itga6	-2.184078616	0.000365418	Down
Itga8	2.148220887	0.00703812	Up
Itgb3	-1.524734672	0.013890878	Down
Itgb6	-3.903786119	0.018342665	Down
Jun	-2.126601206	3.23E-14	Down
Lama1	1.980485668	1.47E-05	Up
Lif	-3.208008356	0.000124803	Down
Mmp3	-1.64941459	0.015703883	Down
Ngf	-1.034815308	0.000182018	Down
Oat	3.147436669	7.73E-07	Up
Prodh	2.566825143	1.73E-05	Up
Thbs2	1.873124974	4.29E-07	Up
Tnfaip3	-1.863561382	0.000183597	Down

Supplementary Figure S1. Relative hepatic mRNA expression of IL-10 and IFN-γ.

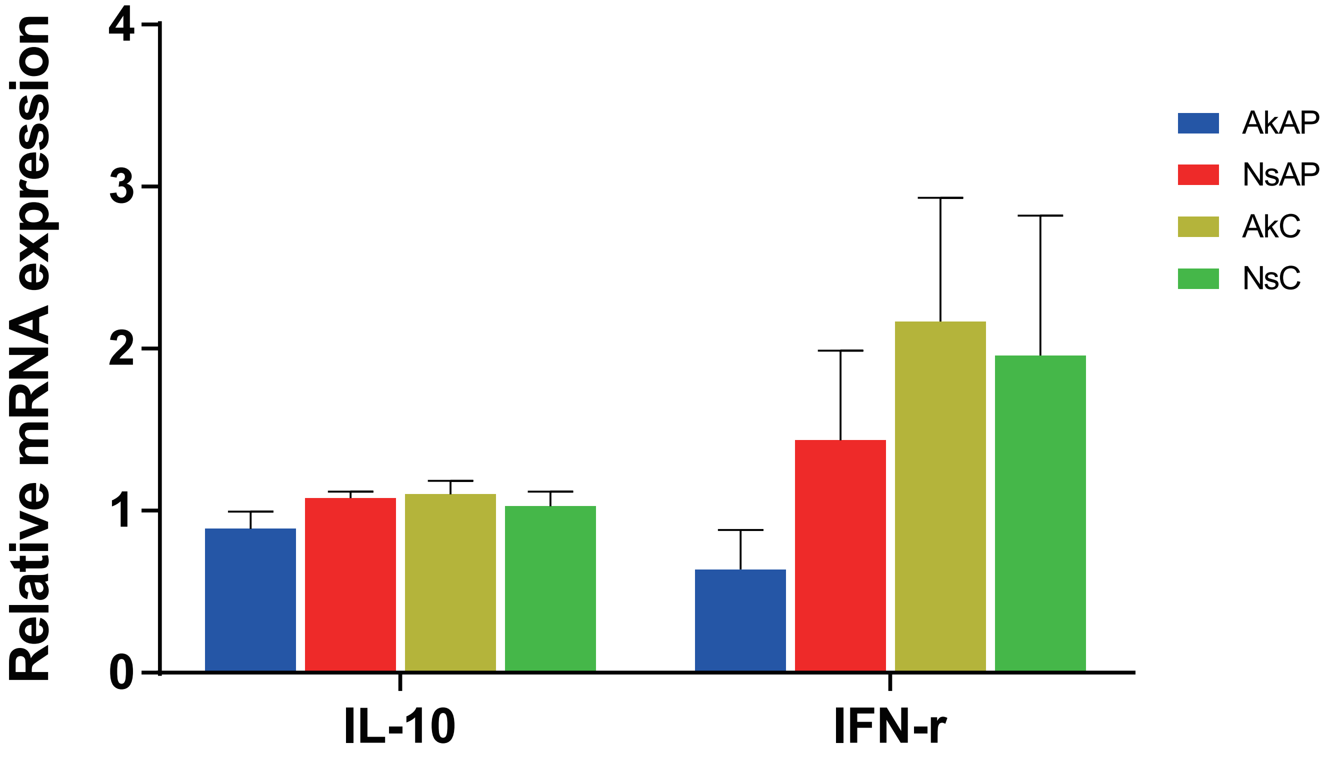

Supplementary Figure S2. Representative flow cytometric plots of macrophages and neutrophils and quantitative analysis of cell percentage

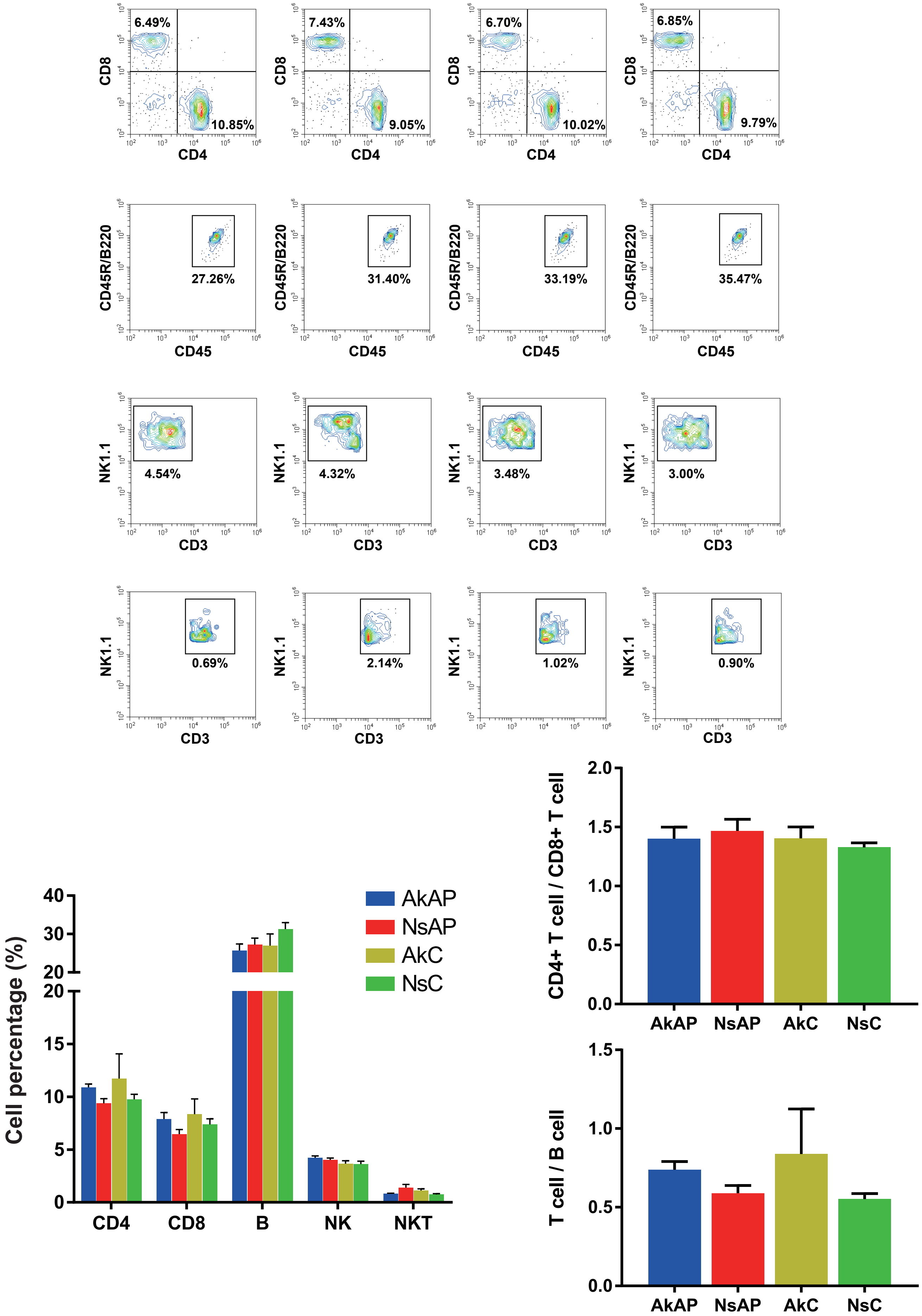

Supplementary Figure S3. Visualization of the cluster 1, cluster 2 and cluster 3 in network.

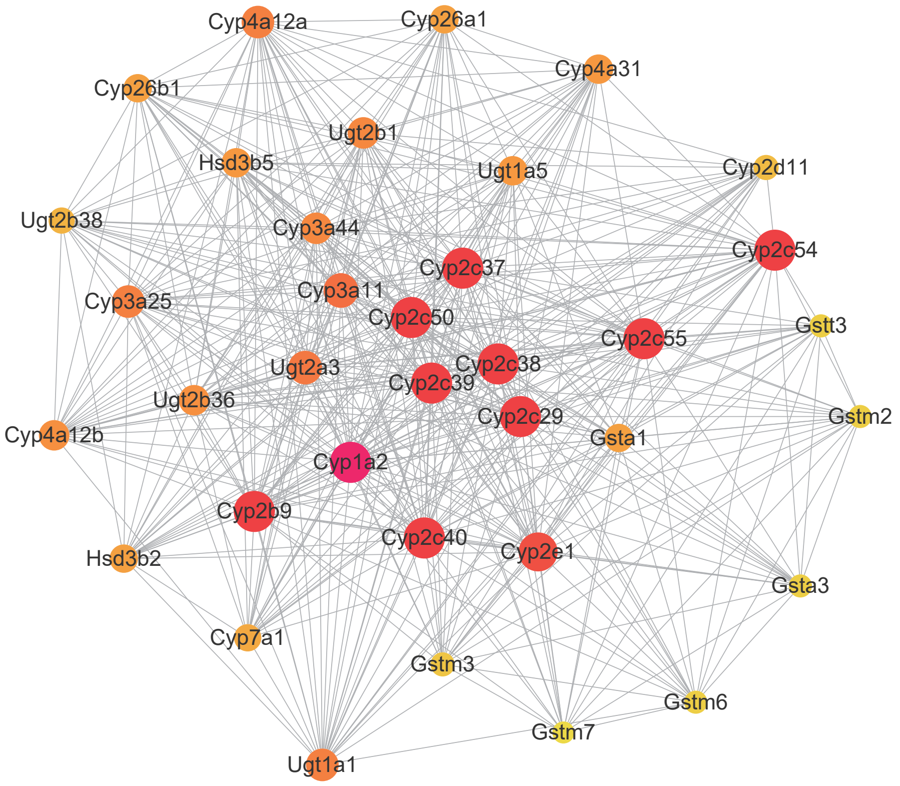

Cluster 1: drug metabolism

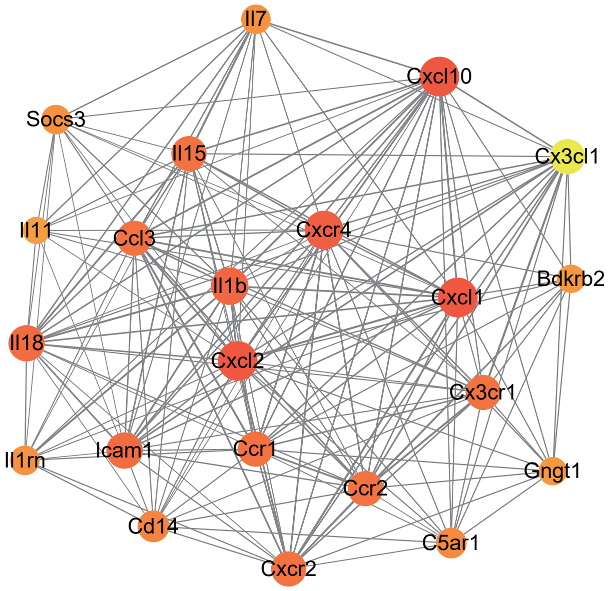

Cluster 2: inflammation

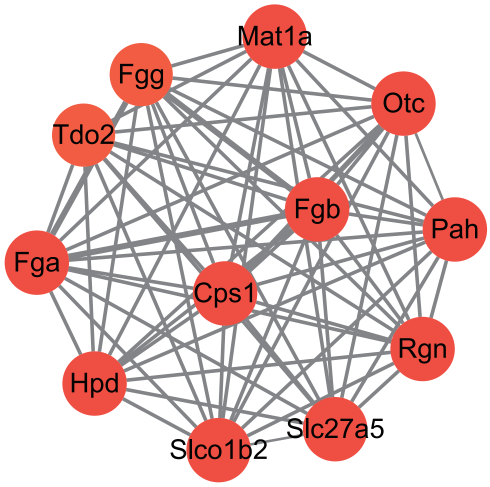

Cluster 3: amino acid biosynthesis

Supplementary Figure S4. The alpha-diversity indexes (Chao1 and Shannon).

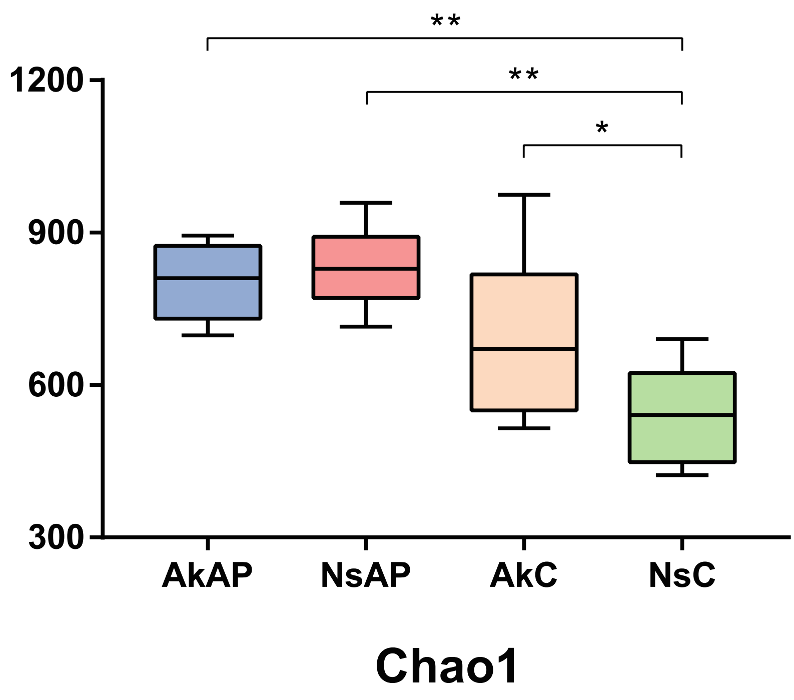

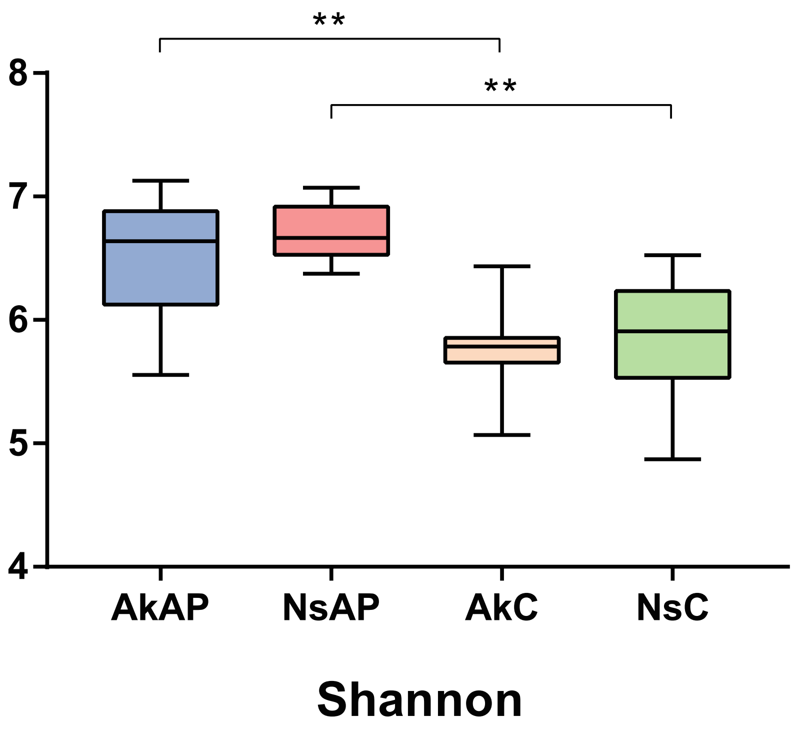

Supplementary Figure S5. Relative abundance of the 10 most abundant taxa at the phyla level.

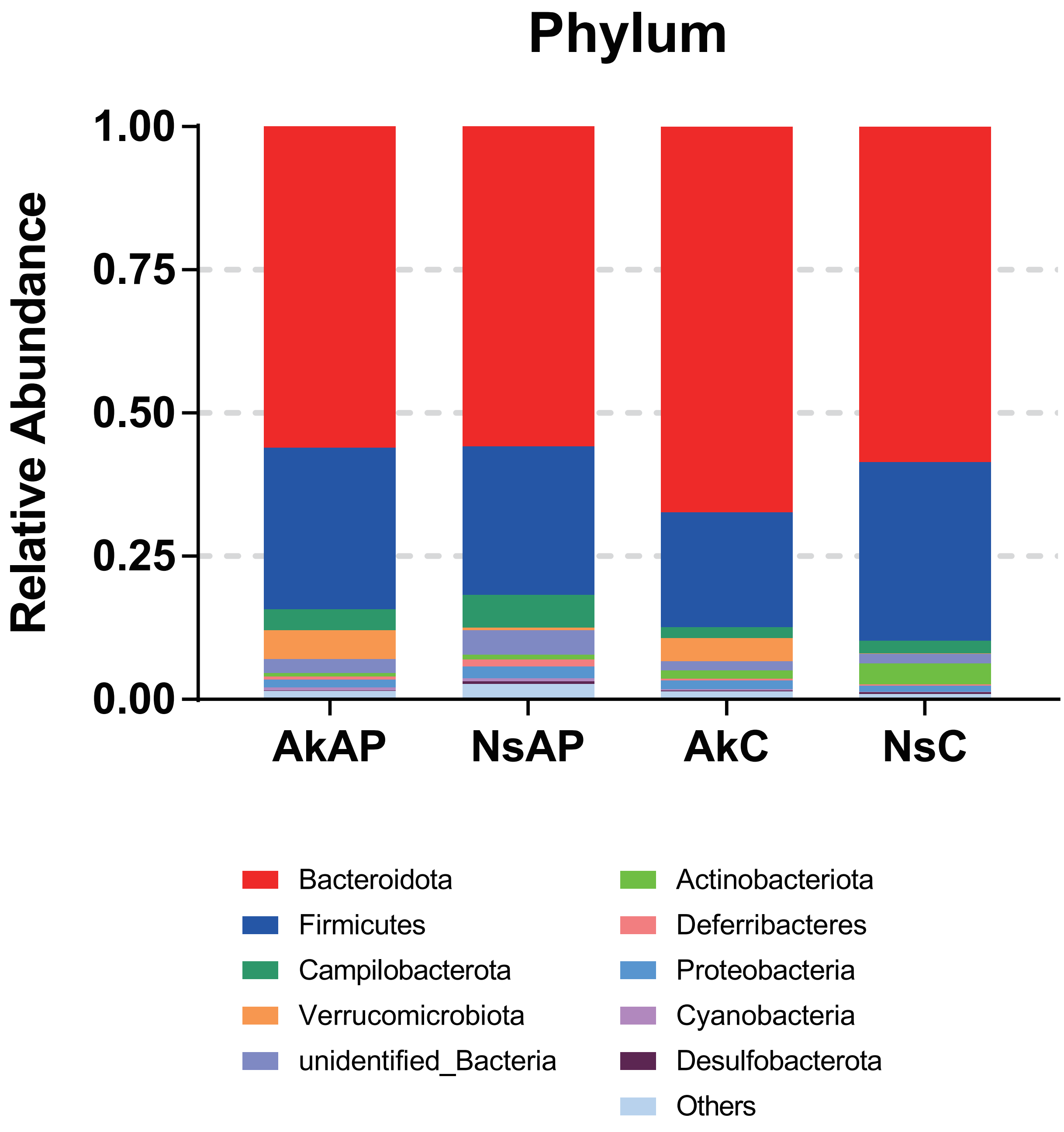

Supplementary Figure S6. Bar chart of the relative abundance of phyla with significant changes (Kruskal-Wallis test). The data are presented as the mean ± SEM. *P < 0.05, **P < 0.01 and ***P < 0.001 for the comparison.

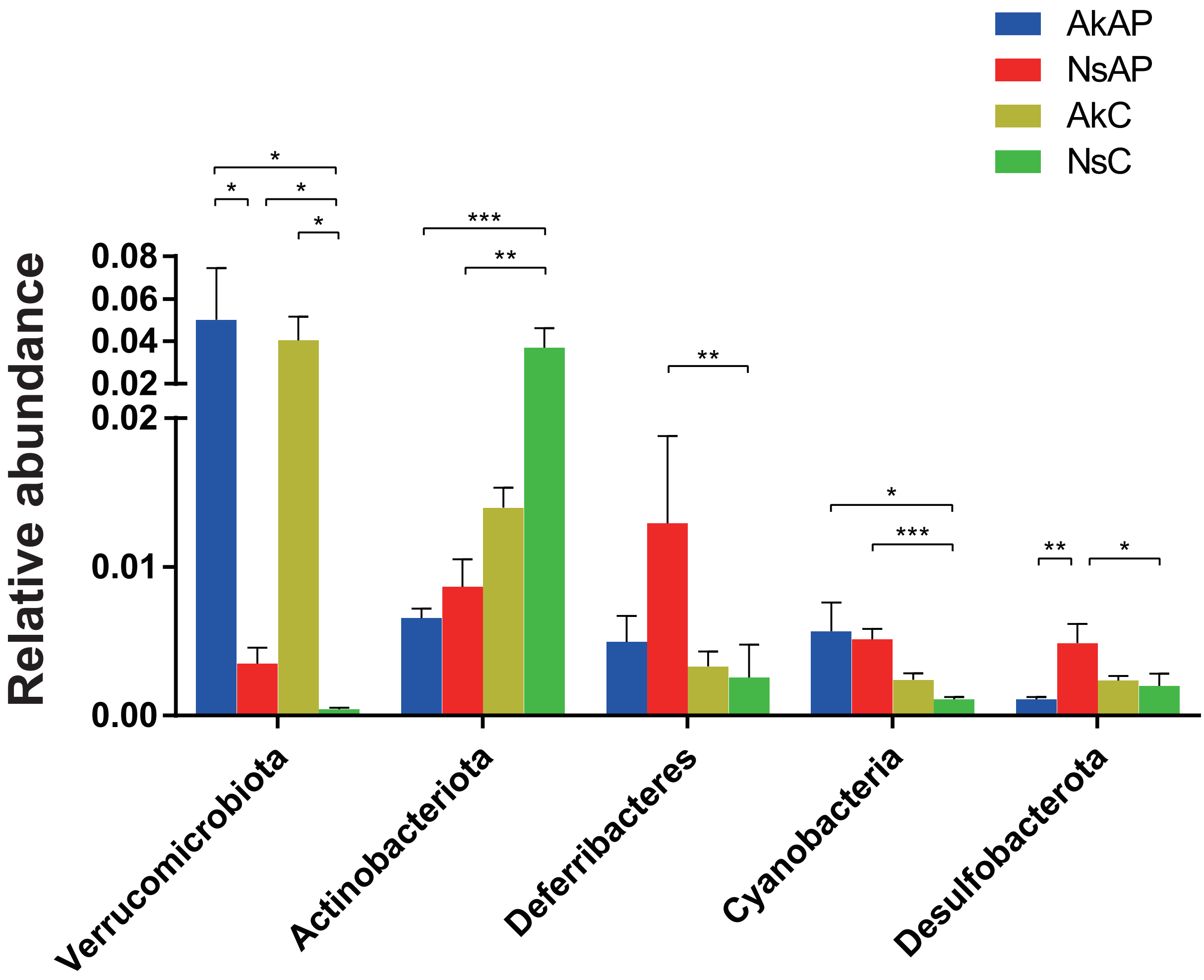

Supplementary Figure S7. Linear discriminant analysis (LDA) effect size (LEfSe) analysis among AkAP, NsAP and NsC. (A) LEfSe cladogram and discriminative taxa with LDA score >3.5 between AkAP and NsAP. (B) LEfSe cladogram and discriminative taxa with LDA score >3.5 between NsAP and NsC.

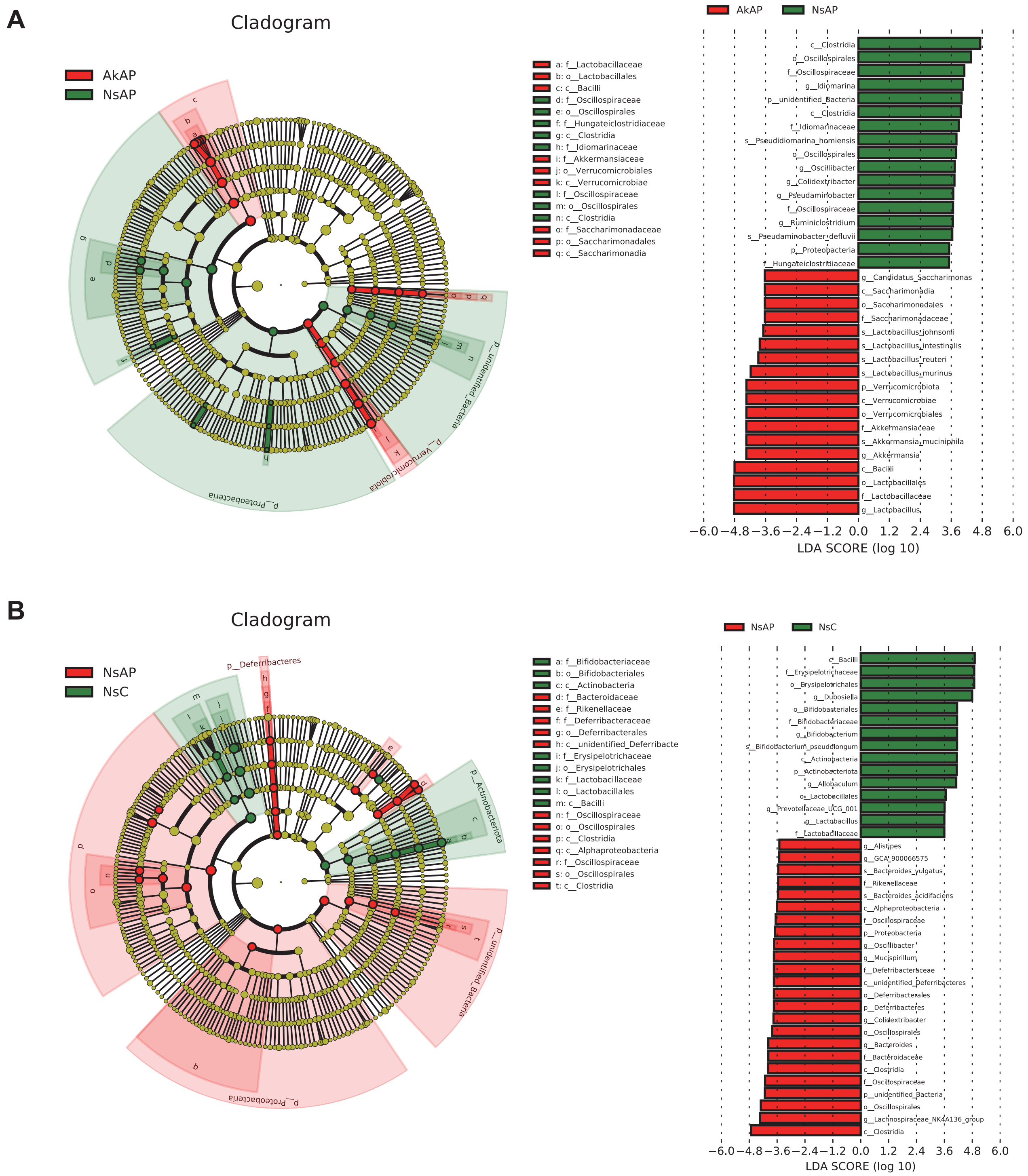

Supplement: SUPPLEMENTAL FILE 1 — Supplemental material. Download SPECTRUM01596-21_Supp_1_seq12.pdf, PDF file, 3.6 MB [file spectrum01596-21_supp_1_seq12.pdf]
